# Glioinflammation: disease-associated microglia and astrocytes in psychiatric disorders, neurodegeneration, and senescence

**DOI:** 10.3389/fncel.2025.1669272

**Published:** 2026-02-17

**Authors:** Kumiko Uemura, Shunya Hiro, Suthinee Attachaipanich, Ruixue Du, Nur Intan Saidaah Mohamed Yusof, Miyu Kinoshita, Momoka Hikosaka, Gen Ohtsuki

**Affiliations:** 1 Department of Neuroscience, Graduate School of Medicine, Kyoto University, Kyoto, Japan; 2 Department of Sports Medicine, Xiangya Hospital, Central South University, Changsha, China; 3 Faculty of Pharmacy, Universiti Teknologi MARA Selangor, Bandar Puncak Alam, Malaysia; 4 Department of Drug Discovery Medicine, Graduate School of Medicine, Kyoto University, Kyoto, Japan

**Keywords:** astrocyte, epigenetics, glial heterogeneity, microglia, mitochondria, neurodegenerative disease, psychiatric disorder, senescence

## Abstract

In this review, we synthesize recent conceptual and experimental advances in neuroscience, highlighting selected studies that delineate the roles of reactive microglia and astrocytes in the contexts of developmental inflammatory stress, neurodegenerative diseases, and cellular senescence. Since the characterization of disease-associated glial phenotypes in 2017, building on earlier pioneering discoveries, we focus here on disease-associated microglia (DAM) and disease-associated astrocyte (DAA) to reassess their contributions to glio-inflammation. It is now recognized that the stress-induced glial states are far from uniform; however, the ontogeny, molecular determinants, and functional consequences of this heterogeneity remain incompletely understood, particularly in psychiatric disorders, Alzheimer's disease, and amyotrophic lateral sclerosis. Accordingly, we compare the glial heterogeneity and its underlying mechanisms across translational mouse models and human neuropathology, considering their evolutionary and physiological contexts. While this review does not aim to be exhaustive, we propose an integrative framework that redefines glial stress responses through the combined lenses of inflammation, transcriptomics, mitochondrial dynamics, lipid metabolism, epigenomic regulation, and cellular senescence. Finally, we outline emerging frontiers for AI-enabled multi-omic physiological and pathological approaches, emphasizing their potential to illuminate glial state transitions and accelerate therapeutic discovery in the near future.

## Introduction

The concept of neuroglia was first proposed by Rudolf Virchow, who coined the term in 1846 and suggested that it served as the “nerve glue” maintaining the structural integrity of the brain. Camillo Golgi conducted detailed studies on glial cells, describing their structural and functional distinctions from neurons, later. Santiago Ramón y Cajal identified protoplasmic astrocytes and referred to glial cells as the “third element” of the nervous system, distinguishing them from both neurons and blood vessels. Pío del Río-Hortega, a student of Cajal, made a major contribution by identifying microglia and defining oligodendrocytes as distinct glial subtypes. After tracing the historical evolution of the concept and function of glial cells (Somjen, 1988), glial cells are now one recognized as the crucial participants in every major aspect of brain development, neural functions, and neurological diseases (Barres, 2008; Paolicelli et al., 2022; Escartin et al., 2021; Labarta-Bajo and Allen, 2025).

### Microglia and astrocytes in brain homeostasis and pathology

**Figure 1:**
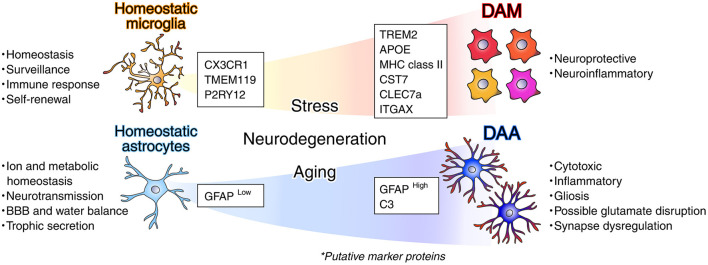
Concept of disease-associated microglia and astrocytes. Microglia are resident immune cells of the CNS, responsible for both host defense and the maintenance of homeostasis. Homeostatic microglia express specific markers (both genes and proteins) for innate immunity, metabolism, and homeostatic regulation. Disease-associated microglia (DAM; Keren-Shaul et al., 2017; Ajami et al., 2018; Mrdjen et al., 2018) also express characteristic markers and functionally separate at least two populations: neuroprotective and neuroinflammatory. The latter has been termed disease-inflammatory microglia, DIM (Silvin et al., 2022). Otherwise, microglial neurodegenerative phenotype (MGnD) has different features (Krasemann et al., 2017). Astrocytes are known to exhibit a reactive form. In their homeostatic state, astrocytes contribute to ion and metabolic balance, support neurotransmission by forming tripartite synapses and by regulating the uptake and synthesis of neurotransmitters, maintain blood-brain barrier (BBB) integrity and water homeostasis, and secrete trophic factors such as BDNF, GDNF, and NGF. Disease-associated astrocytes (DAA; Liddelow et al., 2017; Habib et al., 2020) also express signature markers, including C3 and GFAP, in response to microglial stimulation or neurodegeneration. DAA are associated with cytotoxicity, inflammation, gliosis, and potential glutamate metabolism disruptions and, thus, synaptic dysfunction.

Microglia are the immune cells residing in the brain and spinal cord, monitoring infection and causing stress responses. They originate from the yolk sac in the mother's womb during early development (Ginhoux et al., 2010; Askew et al., 2017; Prinz et al., 2019; Heneka et al., 2025) without any contribution from bone-marrow-derived monocytes to the microglial pool, at least under homeostatic conditions (Bruttger et al., 2015; Masuda et al., 2020), and healthy brain parenchyma has little macrophage and monocyte infiltration due to blood-brain barrier (BBB). Microglia composed 5%−10% of cells in the brain and show heterogeneity and variability across brain regions in their morphology, function, transcriptomes, and proteomes. In human 28% microglia renew per year, and microglial cells are on average 4.2 years old (Réu et al., 2017). Thus, the approximate rate of microglia turnover is 0.08% a day; a low turnover rate in comparison with other immune cells (granulocytes, monocytes, and naïve B cells), despite less turnover of other cells in the central nervous system (CNS; Réu et al., 2017). In 2017, a couple of landmark studies defined disease-associated microglia (DAM; Keren-Shaul et al., 2017; Ajami et al., 2018; Mrdjen et al., 2018), microglial neurodegenerative phenotype (MGnD; Krasemann et al., 2017), and monocyte-derived TREM2-expressing disease inflammatory macrophages (DIM; Silvin et al., 2022) in human specimen and translational murine models (Figure 1, Table 1). Microglial signatures and their role in health and disease were discussed (Butovsky and Weiner, 2018; Martins-Ferreira et al., 2025). Several studies employing single-cell RNA sequencing, mass cytometry, and fluorescence cytometry revealed phenotypic alterations in microglia across various models, although spatial information was limitedly obtained and the pathological mechanisms remained unclear at that time. According to Silvin et al. (2022), embryonically-derived TREM2-dependent neuroprotective DAM and monocyte-derived TREM2-independent disease-inflammatory macrophages (DIMs) accumulate with aging and are conserved in humans. DAM may exhibit a neuroprotective signature, while DIMs are associated with inflammation during neurodegeneration. Therefore, so-called “DAM” is composed of different subpopulations. In this review, we do not classify them to maintain simplicity.

**Table 1:** Disease-associated microglia (DAM) and microglial heterogeneity.

**Year**	**Disease, age, conditionings**	**Animal species**	**Brain region**	**Omics method**	**Feature molecules or clusters**	**Notes**	**References**
2002	AD	Human	Hippocampal CA1	GeneChip Arrays	DAXX, cPLA2, CDP5, NF-κB2 p100/p52, FAS, βAPP, DPP1, NFIL6, IL precursor, B94, HB15, COX-2, and CEX-1	AD hippocampal CA1 showed decreased expression of transcription factors, neurotrophic factors, synaptophysin, metallothionein III, and metal regulatory factor-1. RNA levels of DAXX, cPLA2, CDP5, NF-kappaBp52/p100, FAS, betaAPP, DPP1, NFIL6, IL precursor, B94, HB15, COX-2, and CEX-1 were increased ≥ three-fold.	Colangelo et al., 2002
2007	Aging	Mouse	Whole brain	qRT-PCR	TNFα, IL-1β, IL-6, and IL-10	Aging microglia showed increased expression of TNFα, IL-1β, IL-6, IL-10, and TGFβ1, along with reduced process complexity and lipofuscin accumulation. After LPS challenge, aging microglia maintained a consistent fold-change in cytokine response, indicating functional inflammatory machinery. Basal cytokine levels (TNFα, IL-1β, IL-6, IL-10) were elevated in aging microglia compared to young.	Sierra et al., 2007
2007	AD	Mouse	Whole brain	qRT-PCR	Ccr2	Ccr2 deficiency leads to decreased microglial accumulation in the brain at early stages of AD, and increased Aβ deposition particularly in and around blood vessels. Early microglial accumulation in the AD brain is Ccr2 dependent.	El Khoury et al., 2007
2008	AD	Mouse	Whole brain	qPCR	SRA, CD36, RAGE	In aged PS1-APP mice, microglia showed reduced expression of Aβ-binding receptors (SRA, CD36, RAGE) and degrading enzymes (insulysin, neprilysin, MMP9). IL-1β and TNFα were increased in these microglia. TNFα reduced SRA and CD36 expression and Aβ uptake in cultured microglia.	Hickman et al., 2008
2013	Aging	Mouse	Whole brain	Bulk RNA-seq, proteomics	P2yr12, P2ry13, Adora3, Siglech	More than 81% of the microglial senosome genes, which are in involved in sensing endogenous ligands, were downregulated during aging.	Hickman et al., 2013
2017	AD, ALS, aging	Human, mouse	Whole brain	sc-RNA-seq	[Early] CD9, Itgax (CD11c), Clec7a, and CD63; [Late] Cst7, Lpl, and Trem2	Described a novel microglia type associated with neurodegenerative disease (DAM) and identified markers, spatial localization, and pathways related to this subset.	Keren-Shaul et al., 2017
2017	AD, ALS, MS, aging	Human, mouse	Cortex samples (human); spinal cord and brain (mouse)	Bulk RNA-seq, qRT-PCR, proteomics (LC-MS/MS)	TREM2, APOE	Identified a molecular signature of disease-associated microglia (MGnD) and revealed that this signature is dependent on the TREM2-APOE pathway.	Krasemann et al., 2017
2017	AD	Human, mouse	Frontal cortex and temporal lobes (human); cortex, hippocampus, and whole brain for RNA-seq (mouse)	Bulk RNA-seq	RIPK1, Cst7	RIPK1 is highly expressed in microglia in human AD brains. In APP/PS1 mice, RIPK1 inhibition reduced amyloid burden, inflammation, and memory deficits. RIPK1 regulates microglial CH25H and Cst7 expression, impairing lysosomal Aβ degradation and leading to DAM phenotype.	Ofengeim et al., 2017
2017	AD, FTLD, PD, SCZ	Human, mouse	Cortex	Bulk RNA-seq, ATAC-seq, CHIP-seq	Active genes and enhancers	Examined microglial transcriptomes and epigenetics among human, mouse or after culturing and identified that genes exhibiting different gene expression were often implicated in neurodegenerative disease.	Gosselin et al., 2017
2018	AD	Human, mouse	Frontal cortex and hippocampus	WGCNA (weighted correlation network analysis of microarray-measured samples), qRT-PCR	Pro-inflammatory (e.g., Tlr2, CD44, Kv1.3) and anti-inflammatory (e.g., Igf1, CXCR4)	WGCNA revealed two distinct DAM subtypes in AD mouse models: pro-inflammatory (e.g., Tlr2, CD44, Kv1.3) and anti-inflammatory (e.g., Igf1, CXCR4). LXRα/β agonists and Kv1.3 inhibitor (ShK-223) shifted DAM profiles toward anti-inflammatory phenotypes and enhanced Aβ clearance. Pro-inflammatory DAM markers correlated with AD neuropathology and preceded cognitive decline in human datasets.	Rangaraju et al., 2018
2018	Aging, AD	Human	SH-SY5Y human neuroblastoma culture	-	IL-8, IL-6, IL-6, p-mTOR/T-mTOR, p-eIF2a/T-eIF2a, FMRP, etc.	Iron-overloaded human microglia modeled a senescent phenotype, showing increased ER stress and reduced autophagy. These senescent microglia secreted less insulin-degrading enzyme, leading to elevated extracellular β-amyloid in SH-SY5Y neuron co-culture.	Angelova and Brown, 2018
2020	AD	Human, mouse	Prefrontal cortex (dorsolateral)	sn-RNAseq	DAM genes, Cst7, Lpl and Csf1, in microglia sub-clusters.	Single-nucleus RNA-seq revealed Trem2-dependent DAM and a novel Serpina3n^+^C4b^+^ reactive oligodendrocytes in 5XFAD mice. Human AD showed IRF8-like microglia, impaired oligodendrocyte myelination, and weakened astrocyte–neuron metabolic coupling. TREM2-R47H and R62H variants showed reduced microglial activation.	Zhou et al., 2020
2022	AD	Human (AD vs. non AD) and Mouse	Whole brain	sc-RNA-seq	Murine signature genes of DAM (B2M, CD63, MAMDC2, CCL3, GPNMB, SPP1, TYROBP, and TREM2); DIM (CCL4, CD14, CD83, CSF2RA, EIF1, FOS, IER2, JUN, JUNB, IL1B, TNF, PLAUR, SAT1, and BTG2)	Single-cell RNA-seq integration identified two distinct brain macrophage populations in AD models: embryonic TREM2-dependent neuroprotective DAM and monocyte-derived TREM2^+^ disease inflammatory macrophages (DIMs) accumulating with aging. Ontogeny and function are conserved in humans. DAM exhibit a neuroprotective signature, while DIMs are linked to inflammation during neurodegeneration.	Silvin et al., 2022
2022	Aging	Mouse	Gray matter from the frontal cortex and white matter from the optic tract, medial lemniscus and corpus callosum	sc-RNA-seq	IFN-responsive microglia: IBA1, STAT1, Ifit3, Usp18, Ifi27l2a …	Aging induces CD8^+^ T-cell–dependent, interferon-responsive states in oligodendrocytes and microglia within white matter. Lymphocyte depletion mitigates oligodendrocyte loss, whereas T-cell activation aggravates it, identifying adaptive immune signaling as a key driver of glial degeneration during aging.	Kaya et al., 2022
2024	Early-onset and late-onset AD, MCI, ALS, FTD, PD, PSP, LBD, MS, HD, and stroke	Human	BA4,9,20, anterior watershed, facial motor nucleus, hippocampus, occipital cortex, spinal cord, substantia nigra, thalamus, and temporal neocortex	sn-RNA-seq, sc-RNA-seq, bulk RNA-seq	Human DAM2hi cluster	Single-cell RNA sequencing of 215,680 human microglia from 74 donors revealed subsets defined by oxidative vs. heterocyclic metabolism. Subtypes were linked to antigen presentation, motility, and proliferation, and enriched in neurodegenerative disease susceptibility genes. Camptothecin downregulated disease-enriched microglial signatures and upregulated Alzheimer's disease, associated microglial signatures *in vitro*.	Tuddenham et al., 2024
2025	Developmental disorders (MIA + RSDS)	Human, mouse	Cerebellum, motor cortex, prefrontal cortex, and hippocampus	sc-spatial proteomics (imaging mass cytometry)	SAM proteins: IL6ST, TGFBR2, TREM2, MHC-class II, MMP9, APOE, Ki67, TGF-β1, Caspase-1, and Lyve1	In a mouse model combining maternal infection and social stress (2HIT), microglia increased specifically in the cerebellum and correlated with Purkinje neuron loss. Imaging mass cytometry and single-cell proteomics identified a transition to TREM2^+^ stress-associated microglia (SAM) linked to IL-6 and TGFβ signaling. Microglia replacement, both systemic and cerebellum-targeted, ameliorated cerebellar dysfunctions. Aged human sample also showed SAM-like cells.	Hikosaka et al., 2025
2025	AD, MS, ASD, LBD, epilepsy, COVID-19	Human		snRNA-seq/scRNA-seq datasets	DAMs (clusters 1, 3, 5, 6), and DIMs (cluster 2) populations	The Human Microglia Atlas (HuMicA), integrating 19 snRNA-seq/scRNA-seq datasets from 241 samples across 7 conditions, defined 9 microglial populations. Four subtypes of disease-associated microglia and disease-inflammatory macrophages were identified and found across AD, autism, MS, and others. A GPNMB-high microglial subpopulation was expanded in AD and MS.	Martins-Ferreira et al., 2025
2025	Aging, MS	Mouse	Spinal cords	Real Time qPCR, bulk RNA-seq	Senescent-like microglia	Senescent microglia and macrophages accumulate in demyelinated lesions with age, sustaining a senescence-associated secretory phenotype that impairs remyelination. Elevated CCL11/Eotaxin-1 within the SASP inhibits oligodendrocyte maturation, while senolytic treatment restores myelin regeneration in younger but not aged mice.	Gross et al., 2025

This table summarizes recent studies investigating DAM and microglial heterogeneity. Studies are organized by publication year, brain region, disease, age, experimental conditions, species, omics techniques, characteristic molecules, key observations, and references.

AD, Alzheimer's disease; ALS, amyotrophic lateral sclerosis; ASD, autism spectrum disorder; COVID-19, coronavirus disease 2019; FTD, frontotemporal dementia; FTLD, frontotemporal lobar degeneration; HD, Huntington's disease; LBD, Lewy body dementia; MCI, mild cognitive impairment; MIA, maternal immune activation; MS, multiple sclerosis; PD, Parkinson's disease; RSDS, repeated social defeat stress; PSP, progressive supranuclear palsy; SCZ, schizophrenia; sc-RNAseq, single-cell RNA sequencing; sn-RNAseq, single-nucleus RNA sequencing.

As was previously discussed, it was unclear whether peripheral macrophages would eventually differentiate into bona fide microglia and thereby assume the exact physiological roles of microglia in the CNS. Cronk et al. (2018) reported that brain-engrafting macrophages are a unique cell type which, although capable of taking up long-term residence in the CNS, retain a distinct transcriptional and functional identity (Cronk et al., 2018). During CNS inflammation, the microglial pool is thought to be partially reshaped by bone marrow-derived macrophages or monocytes during aging and in disease conditions such as ischemia, glioma, multiple sclerosis, amyotrophic lateral sclerosis (ALS), and Alzheimer's disease (AD; Ritzel et al., 2015; Vogel et al., 2013; Silvin et al., 2023; Quek et al., 2022; Monoranu et al., 2021). However, it remains unclear whether infiltrating macrophages become long-term residents in the human brain or perivascular space and how they are reprogrammed (Silvin et al., 2023; Heneka et al., 2025). Along to human studies, it is important to address at least the following challenges: the availability of human post-mortem tissue through brain banks or living tissue/samples and the limitations related to its use; the post-mortem interval of samples; the technical tools currently available; the neuroimmune aspects that need to be explored and validated in the human brain; and the experimental observations derived from animal models. These factors make it difficult to achieve a complete understanding of DAM characteristics.

Meanwhile, astrocytes play essential roles in maintaining homeostasis, regulating metabolism, development, and modulating synaptic transmission in the CNS. Astrocytes are namely star-like shaped glial cells that support neurons and modulate synaptic activity, typically by producing glutamate. Astrocytes also establish functional, metabolic, and physical contacts with surrounding cells, including neighboring astrocytes, neurons, and endothelial cells of blood vessels. Those interactions have been historically shown to allow them to Ca^2+^ homeostasis, metabolic, and supportive function in the brain (Nedergaard, 1994; Cotrina and Nedergaard, 2002). The contribution of reactive astrocytes to neurodegenerative diseases and cognitive impairments is now well established. Increasing evidence of astrocyte dysfunction in disease pathology has spurred interest in developing astrocyte-targeted therapeutic strategies (Figure 1, Table 2; Barres, 2008; Cahoy et al., 2008; Zamanian et al., 2012; Vartak-Sharma and Ghorpade, 2012; Liddelow et al., 2017; Sadick and Liddelow, 2019; Habib et al., 2020; Hasel et al., 2021). The proportion of glial cells (i.e., astrocytes, microglia, and oligodendrocytes) against neurons is no longer considered simply 10:1 (Herculano-Houzel, 2014; von Bartheld et al., 2016). According to Andrade-Moraes et al. (2013), the human glia-to-neuron ratio in the cerebral cortex excluding white matter ranges from 1.15 to 1.64. More specifically, non-neuronal/neuronal ratios in the gray matter of the entire cerebral cortex is 1.6 in control and 3.4 in AD, respectively. Interestingly, the glia-to-neuron ratio remains relatively constant across species, irrespective of brain or cellular size, but is strongly correlated with neuronal density throughout evolution (Herculano-Houzel, 2014). Astrocytes are roughly 10% in rodents (Nimmerjahn et al., 2004), 30% in rhesus monkey (Peters et al., 2008), and 20%−40% in humans (Pelvig et al., 2008) of the total glial population, with both developmental and regional differences.

**Table 2:** Disease-associated astrocytes (DAAs) and reactive astrocytes.

**Year**	**Disease, age, conditionings**	**Animal species**	**Brain region**	**Omics method**	**Feature molecules or clusters**	**Notes**	**References**
2008	Development	Mouse	Forebrain	GeneChip Arrays		FACS-purified astrocytes from mouse forebrains across development showed Aldh1L1 a specific astrocyte marker than GFAP. Astrocytes showed enrichment in metabolic, lipid synthesis, and phagocytic pathways like Megf10 and Mertk.	Cahoy et al., 2008
2012	Ischemia (MCAO), infection (LPS)	Mouse	Neocortex, striatum, and hippocampus	GeneChip Arrays (mRNA)	Lcn2, Serpina3n	Gene expression profiling revealed that reactive gliosis involved Lcn2 and Serpina3n gene expression as strong markers of reactive astrocytes in ischemic stroke or neuroinflammation by LPS.	Zamanian et al., 2012
2012	AD	Mouse	Hippocampus	-		Using AAV-Gfa2-VIVIT to inhibit calcineurin/NFAT pathway in astrocytes of APP/PS1 mice reduced astrocyte activation. Treatment led to improved cognition, synaptic function, and reduced amyloid and glial markers.	Furman et al., 2012
2012	Injury (wound-healing model)	Human, mouse		-	AEG-1	The study identified astrocyte elevated gene-1 (AEG-1), a human immunodeficiency virus 1 or tumor necrosis factor α-inducible oncogene, in regulating astrocyte responses to injury.	Vartak-Sharma and Ghorpade, 2012
2016	Aging	Human	Human fatal astrocyte culture	qRT-PCR		Senescent astrocytes upregulated pro-inflammatory genes (p21, IL-8, IL-12, cyclin D1, ICAM-1, IGFBP-5, and CXCL12). Senescent astrocytes also showed reduced expression of genes involved in astrocyte-identification, development and antigen presentation (GFAP, S100b, ALDH1L1, FGFR3, and SYNDIG1).	Crowe et al., 2016
2017	MCAO, AD, HD, PD, ALS, and MS	Human, rat, mouse	Hippocampus and prefrontal cortex	qRT-PCR	Il-1α, TNF, and C1q	A1 reactive astrocytes are induced by activated microglia via Il-1α, TNF, and C1q, A1 astrocytes lose supportive functions and actively kill neurons and oligodendrocytes in human AD and ALS.	Liddelow et al., 2017
2018	Aging	Mouse	Visual cortex, motor cortex, cerebellum, and hypothalamus	Bulk RNA-seq	UP: Gfap, C4b, Serpina3n, Sparc, Pcdhb6,11, caspase 1,12, etc. DOWN: Bmp4, Bmp6, Tnc.	Aging astrocytes showed region-specific and shared gene expression changes across the brain. Homeostatic and neurotransmission-related genes remain largely stable with age. Genes promoting synapse elimination are upregulated.	Boisvert et al., 2018
2018	Aging	Mouse	Striatum, hippocampus, and neocortex	Astrocyte-specific RNAseq	Il-1α, TNF, and C1q	Hippocampal and striatal astrocytes upregulate more reactive genes than cortical astrocytes with age. Microglial cytokines IL-1α, TNF, and C1q drive A1 astrocyte formation during aging.	Clarke et al., 2018
2018	ALS	Human, rat	Spinal cord, brain, and CSF	qRT-PCR	BMP4	BMP4 was upregulated and noggin downregulated in reactive astrocytes of ALS rats. BMP4 knockdown via antisense oligonucleotides similarly suppressed glial activation.	Shijo et al., 2018
2018	ALS	Mouse, rat	Spinal cord and CSF	-	miR-218	Motor neuron-derived miR-218 is released extracellularly in ALS. miR-218 suppresses astrocytic EAAT2 expression and downregulates additional targets in ALS pathology. Blocking miR-218 in ALS mice rescued EAAT2 levels and astrocytes.	Hoye et al., 2018
2019	AD	Human	Prefrontal cortex	sn-RNA-seq	AD-pathology-associated astrocyte: GLUL and CLU	Single-nucleus RNA-seq revealed AD subpopulations, involving myelination, inflammation, and neuron survival regulators. Early changes were cell-type specific; late-stage genes reflected shared stress responses. Female cells showed enrichment in disease-related subpopulations with sex-specific transcriptional patterns.	Mathys et al., 2019
2020	AD, Aging	Human, mouse	Hippocampus and prefrontal cortex	sn-RNA-seq	SerpinA3N	Single-nucleus RNA-seq revealed a disease-associated astrocyte (DAA) state that emerges early and expands with AD progression. DAAs also appear with aging in WT mice and humans, clustering near amyloid plaques and expressing inflammatory markers like SerpinA3N. DAAs and DAMs share gene signatures.	Habib et al., 2020
2020	AD	Human, mouse	Prefrontal cortex (dorsolateral)	sn-RNAseq	UP: Gfap, C4b, NCAN, COL5A3. DOWN: FABP5, HILPDA, SOD2.	(Same as in Table 1)	Zhou et al., 2020
2021	LPS-inflammation	Mouse	Across brain regions in a coronal section	sc-RNA-seq, spatial RNA-seq	Apoe, Gfap, Aqp4, Slc1a3. Inflammation: Igtp, Ifit3 and Iigp1.	Single-cell RNA-seq of astrocytes after LPS-induced inflammation showed distinct inflammatory astrocyte subtypes with defined gene expression profiles. Spatial transcriptomics linked specific reactive astrocyte sub-states to defined brain regions.	Hasel et al., 2021
2022	ALS	Human	iAstrocytes (induced-astrocytes)	-	GFAP, CX43, Ki-67, miR-155 and miR-146a	iAstrocytes from ALS patients showed neurotoxicity and stratified by markers including GFAP, CX43, and miR-146a. miR-146a levels in iAstrocytes and the sEVs varied among patients. Restoring miR-146a in depleted iAstrocytes reversed their inflammatory state.	Gomes et al., 2022
2022	AD	Mouse	Cerebellum, dorsal spinal cord, hindbrain, hippocampus, hypothalamus, midbrain, motor, somatosensory, visual cortices, striatum, thalamus, and olfactory bulb	Astrocyte-specific RNAseq	*in notes*	Region-specific astrocyte gene networks were mapped across the mouse CNS, revealing diverse functions and morphologies. Several morphology-linked gene networks included AD risk genes: Aldh1l1 and Sox9, Kcnj10, Slc1a2, Apoe, Kcnj10, Kcnj16, Atp1a2, Gpr37l1, S1pr1, Ntsr2, Ednrb, Smo, Adora2b, Olfr287, Gpr146, Agtrap, Fzd1, Fzd9, and Npr2.	Endo et al., 2022
2023	AD	Mouse	Dorsal preoptic brain region	Bulk RNA-seq	Kcnj2, C4b, Ddr1, and Gfad	High-fat diet (HFD) induces gene expression changes in astrocytes and microglia similar in AD. C4b, upregulated in both HFD and AD, is specifically expressed in astrocytes and colocalizes with GABAergic neurons. Single-cell and spatial transcriptomics showed a potential astrocyte-neuron interaction with C4b and Gad2.	Lin L. et al., 2023
2023	AD	Human, mouse	Whole brain	Bulk RNA-seq	DAA genes: Aqp4, C4b, VIM, CTSB, OSMR, and BAG3.	Astrocytic Bag3 (autophagy chaperone) overexpression reduces alpha-synuclein spreading in mice. BAG3 is expressed in DAAs in human AD.	Sheehan et al., 2023
2024	ALS, FTLD	Human	Motor cortex and prefrontal cortex	sc-RNA-seq	DAA cluster	Single-cell transcriptomics of motor and prefrontal cortices revealed shared molecular signatures in vulnerable layer 5 neurons across ALS and FTLD. Motor and spindle neurons showed nearly identical transcriptional profiles.	Pineda et al., 2024
2024	AD	Mouse	Cortex, hippocampus, and midbrain	Spatial transcriptomics (GeoMx)	*in notes*	Ozone (O_3_) exposure in 5xFAD mice increased astrocyte and plaque numbers, enhanced DAA gene expression (Ctss, Sparc, Fcgr3, Cx3cr1, Mpeg1, Serpina3n, Laptm5, Apoe, Csf1r, C1qb, Itgb5, Tyrobp, Lag3, C1qa, Cd63, and Trem2), and altered astrocyte-microglia interactions. O_3_ impaired the astrocyte response to plaque localization. Hmgb1^fl/fl^ LysM-Cre^+^ mice showed loss of peripheral myeloid HMGB1 and dysregulated transcriptomic profiles.	Ahmed et al., 2024
2024	AD	Human, mouse	Entorhinal cortex and prefrontal cortex	Meta analysis (sn-RNA-seq and bulk RNA-seq data), qRT-PCR	ZEP36L, AEBP1, WWTR1, PHYHD1, DST and RASL12	Six genes (including WWTR1, ZFP36L, AEBP1) were associated with AD severity and validated in 5xFAD mouse models.	Yu et al., 2024
2025	MDD	Human, mouse	Hippocampus (mouse) and blood sample (human)	sc-RNA-seq	CCR5 and CCL5	CCR5^+^ neutrophils were elevated in depressed patients and infiltrated the hippocampus in a mouse depression model. Astrocyte-derived CCL5 was identified as the chemokine driving CCR5^+^ neutrophil infiltration.	Yao et al., 2025

This table summarizes recent studies investigating DAA and astrocytic heterogeneity. Studies are organized by publication year, brain region, disease, age, experimental conditions, species, omics techniques, characteristic molecules, key observations, and references.

AD, Alzheimer's disease; ALS, amyotrophic lateral sclerosis; ASD, autism spectrum disorder; COVID-19, coronavirus disease 2019; FTLD, frontotemporal lobar degeneration; HD, Huntington's disease; LBD, Lewy body dementia; LPS, lipopolysaccharide; MCAO, middle cerebral artery occlusion; MDD, Major depressive disorder; MS, multiple sclerosis; PD, Parkinson's disease; sc-RNAseq, single-cell RNA sequencing; sn-RNAseq, single-nucleus RNA sequencing.

### Conceptual shift to disease-associated glial cells

Increased brain inflammation, driven by elevated cytokine levels, is commonly observed in psychiatric disorders (Kronfol and Remick, 2000; Yirmiya et al., 2015; Zhang et al., 2023), neurodegenerative diseases, and the aging brains (Michaud et al., 2013; Colonna and Butovsky, 2017; Porcher et al., 2021). Astrocytes are capable of releasing a variety of cytokines, and they are known to contribute to neuroinflammatory responses and disease progression (Barres, 2008; Sadick and Liddelow, 2019). Given their multifaceted involvement in CNS pathology, a reactive form of disease-associated astrocytes has emerged as promising, but technically challenging, targets for therapeutic intervention (Liddelow et al., 2017; Sadick and Liddelow, 2019; Hasel et al., 2021). A major obstacle is the difficulty of selectively targeting disease-relevant astrocyte subpopulations in the absence of well-defined marker proteins and robust methodologies against spatially restricted areas in various states in the disease progression (Liddelow et al., 2017; Habib et al., 2020; Mallach et al., 2024). Reactive astrocytes have been shown to exert cytotoxicity, and as their activation intensifies, astrogliosis contributes to pathological features such as fibril accumulation, vascular damage, and BBB disruption (Liddelow et al., 2017; Segawa et al., 2021). Moreover, astrocytes play an essential role in glutamate homeostasis through the glutamine-glutamate cycle; consequently, astrocyte dysfunction can impair synaptic transmission, induce excitotoxicity, and cognitive and motor impairments (Bindocci et al., 2017; Dodge et al., 2013). Thus, the identification of glial subsets involved in disease initiation, progression, and maintenance remains a critical goal in their molecular characterization.

Until now our interpretations have made it known that glial cells, once considered passive support, are now seen as active coordinators of brain function and disease progression. Microglia and astrocytes reactively shape various compositions between neural circuits and immunity, with their dysfunction and interaction implicated in psychiatric disorders, neurodegeneration, and senescence. Surprisingly, advances in comprehensive transcriptomic profiling have drastically opened the door to a new field, evoking previous comparisons to the fascinating era of cellular neurophysiology and neuroplasticity research. However, a complete understanding of the dynamic interactions and integration between the multiple “disciplines”—brain physiology and glial neurochemistry—remains an unmet challenge. In this review, we are likely to focus discussion on the transition of microglia and astrocytes along with (1) the developmental psychiatric stress; (2) neurodegenerative disease; and (3) cellular senescence, both of which include chronic inflammatory stress but harboring each context. We aim to pose a perspective for targeting the disease-associated glia to ameliorate the symptoms and revert the disease phenotypes and senescence for human wellness.

## Evolutional glial heterogeneity origins and brain function involvement

**Figure 2:**
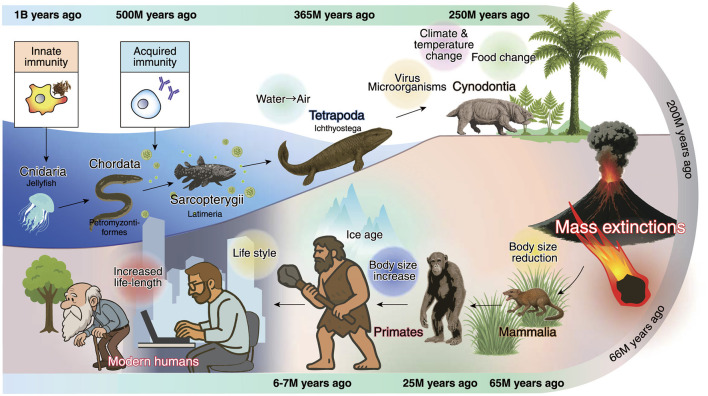
Evolutionary timeline of the vertebrate immune system and mammalian adaptations. Primitive innate immune system emerged more than 1 billion (B) years ago. Agnatha, jawless vertebrates (Chordata), emerged during the late Cambrian period of the Paleozoic era [approximately 520–505 million (M) years ago]. Prior to their emergence, there is no evidence of organisms possessing an adaptive immune system, as defined in vertebrates. During the Devonian period of the Paleozoic era, certain fish equipped with robust fins (early Tetrapoda) began to make the transition onto land. Eventually, cynodonts—direct ancestors of mammals—emerged and survived numerous environmental challenges, including climatic shifts, dietary changes, starvation, infectious diseases, and multiple mass extinction events. After the Cretaceous-Paleogene extinction event (approximately 66 M years ago), mammals underwent an evolutionary trend toward smaller body sizes and shorter lifespans, enabling rapid reproduction but increasing the survival chance. From small early primates such as Purgatorius, body size increased nearly a thousandfold over time, eventually giving rise to great apes and, ultimately, modern humans. Today, advancements in medicine and stable food supplies have allowed humans to achieve both longevities, often exceeding 100 years, and unprecedented levels of prosperity. In other words, human evolution may not anticipate aging in its current biological and societal context.

The immune system plays a central role in host defense by recognizing and eliminating non-self-entities. Macrophages serve as a bridge between innate and adaptive immunity. To understand why microglia are so heterogeneous, related to multiple diseases and aging, it would be important to discern how glial cells emerged in our ancestors. For instance, innate immunity is a fundamental and evolutionarily conserved mechanism found in a wide range of organisms (Figure 2), including cnidarians such as jellyfish and sea anemones, as well as insects and plants. Through the recognition of self and non-self, immune cells can identify and exhibit responses against pathogenic invaders such as bacteria and viruses (Hemmi et al., 2000; Seth et al., 2006). The molecular underpinnings of innate immunity trace back more than a billion years to the last common ancestor of eukaryotes (Figure 2). As multicellular organisms evolved, so too did the sophistication of mechanisms for pathogen discrimination and elimination. Of note, some cnidarians are considered biologically immortal (e.g., *Turritopsis dohrnii* and Hydra). *Turritopsis dohrnii* transdifferentiates itself, allowing rejuvenation, while Hydra continuously regenerates stem cells with high telomerase activity, thereby avoiding cellular senescence. The more elaborate an organism's immune system becomes through evolution, the more prone it is to functional breakdown with age, making the signs of chronic inflammation and aging increasingly apparent.

In contrast, the refinement of neural function fundamentally relies on both divergent and convergent circuit architectures. The emergence of sophisticated visual systems occurred after the Cambrian explosion, approximately 550 million years ago. During development, activity-dependent synaptic pruning via phagocytosis between retinal ganglion cells and thalamic neurons plays a pivotal role in establishing direction selectivity, as characterized in studies by Chen and Regehr (2000). In parallel, microglia mediate the engulfment of supernumerary synapses during postnatal development, revealing an immune [microglia-specific complement receptor 3 (CR3)/C3 signaling]-driven mechanism for sculpting neural circuits (Stevens et al., 2007; Schafer et al., 2012). The cerebellar development emerged after the divergence of jawless vertebrates such as lampreys (Grillner and El Manira, 2020). The cerebellum also undergoes microglia-dependent pruning of surplus climbing fiber synapses for refined motor system (Nakayama et al., 2018). These observations imply that the increasing selective pressure for refined visuomotor control and sensorimotor nervous system during the Cambrian period necessitated the emergence of glia-mediated innate immune mechanisms to ensure their functional precision and elaboration.

Adaptive immunity, by contrast, is defined by its ability to generate antigen-specific responses and immunological memory, providing robust protection upon re-exposure to previously encountered pathogens (Wang R. et al., 2020; Lam et al., 2024). This system is orchestrated primarily by T and B lymphocytes in vertebrates, while invertebrates lack adaptive immunity. Although macrophages are historically regarded as belonging to the innate immune system, they also play indispensable roles in adaptive immunity. Macrophages function as antigen-presenting cells by degrading pathogens and present antigenic fragments on MHC class II molecules to activate CD4^+^ T helper cells. Additionally, they secrete cytokines that shape the differentiation of T cell subsets, including Th1, Th2, Th17, and regulatory T (Treg) cells. The emergence of jawed vertebrates (gnathostomes) approximately 450 million years ago marked the first appearance of the full molecular repertoire of the adaptive immune system, including T and B cells, antibodies, and MHC molecules (Flajnik and Kasahara, 2010; Figure 2). Subsequently, these elements became foundational features of all vertebrate immune systems. Around 500 million years ago, coinciding with the colonization of terrestrial environments and the advent of a more complex nervous system during the Cambrian–Devonian periods, microglia and oligodendrocyte lineages are thought to have emerged (Freeman and Rowitch, 2013). More recently, accumulating evidence suggests that dysregulation of the immune system, once thought to function solely in host defense, contributes to the pathophysiology of neuropsychiatric disorders and neurodegenerative diseases (Figure 2).

According to Hartenstein and Giangrande (2018), astrocytes and oligodendrocytes are neuroectodermally derived lineages that are absent in prebilaterian animals (such as Cnidaria and Ctenophora) and in basal branches of the Bilateria; however, these glial cell types are present in other groups: Molluscs, Annelids, Arthropods, and Chordates. Similarly, dedicated macrophages of the CNS and microglia-like cells are present in annelids and in vertebrates (Figure 2). It is suggestive that glial cells may have evolved multiple times independently but orchestrated with neurons. This evolutionary divergence likely underlies the remarkable variability observed in glial cell transcriptomic and proteomic profiles, morphology, and function. Consequently, glial cells are currently categorized using diverse terminology and classified into numerous subtypes to reflect their biological heterogeneity in response to various stress (Hartenstein and Giangrande, 2018).

Indeed, mammals had to develop adaptive mechanisms to withstand dynamic biotic and abiotic pressures in response to global scale environmental changes. Many mammals evolved smaller body sizes as an adaptation to ecological stresses, such as food scarcity, enabling faster reproduction that promoted species survival and evolution. In contrast, modern humans developed unique lifestyles and technologies that enabled an unprecedented extension of lifespan (Figure 2). As a result, diverse glial immune responses and microbiota configurations may have emerged, leaving lasting biological imprints that could represent the evolutionary origins of aging and disease. The rapid extension of human lifespan, with more individuals living beyond a century, suggests that our evolutionary adaptations may remain suboptimized for sustaining longevity.

## Microglial surveillance and response mechanisms in the healthy and damaged brain

**Figure 3:**
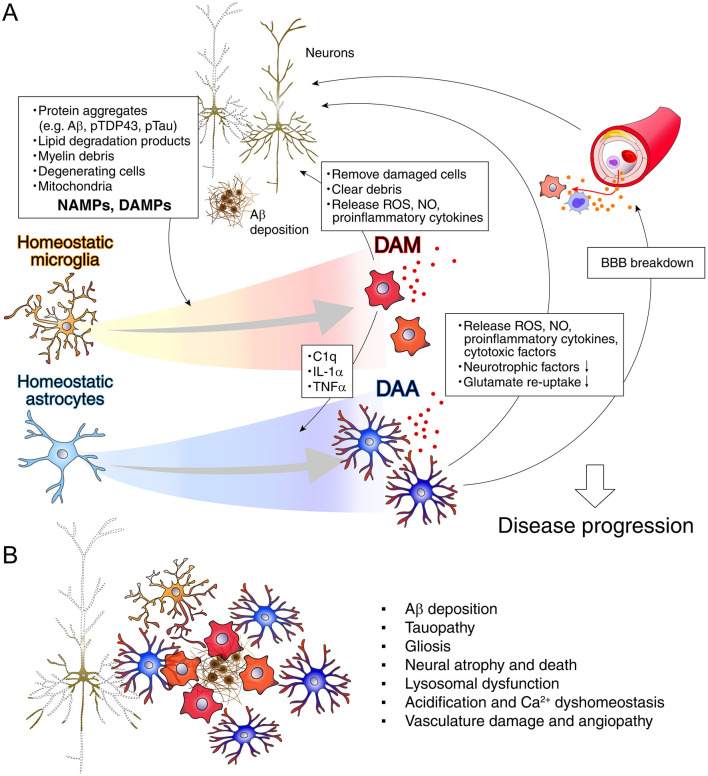
Roles of disease-associated microglia and astrocytes in neurodegenerative diseases. **(A)** Inflammatory stress, neurodegeneration, and aging are accompanied by activation of disease-associated microglia (DAM) and astrocytes (DAA), which release cytokines, cytotoxic factors, and reactive oxygen species. These contribute to damage in neurons, oligodendrocytes, and the vascular system, though microglial and astrocytic states are not uniform. Protein aggregates such as Aβ, phosphorylated TDP-43 (pTDP-43), and Tau further promote inflammation, disrupt mitochondrial function, and trigger neuronal death. Degenerating neurons release neurodegeneration-associated molecular patterns (NAMPs), consisting of cellular debris, myelin fragments, and lipid degradation products, or damage-associated molecular patterns (DAMPs), which drive the shift of microglia toward a reactive state. **(B)** A representative schematic illustrating the cellular architecture of the plaque niche in Alzheimer's disease (Mallach et al., 2024). Key pathological features investigated and reviewed include Aβ deposition (Spires-Jones and Hyman, 2014), tauopathy (Busche and Hyman, 2020), gliosis, neuronal atrophy and death (De Strooper and Karran, 2016), lysosomal dysfunction (Nixon and Rubinsztein, 2024), acidification and Ca^2+^ dyshomeostasis (LaFerla, 2002), and vascular damage and angiopathy (Zlokovic, 2011).

In healthy brains, microglia continuously sense the alterations in CNS microenvironment by extending and retracting their processes. As per the innate immune system, microglia are sensitive to pathogen infections, damage-associated molecular patterns (DAMPs), and peripherally produced neurotoxins to maintain CNS homeostasis (Figure 3). Neuronal injury disrupts Ca^2+^ transients and ATP release as guidance cues for the migration of P2Y12-expressing microglia, related to learning and cognition (Nimmerjahn et al., 2005; Davalos et al., 2005; Parkhurst et al., 2013; Bernier et al., 2019; Tufail et al., 2017).

DAMPs are host-derived molecules that trigger and sustain non-infectious inflammatory responses (Figure 3). They are typically released from damaged or dying cells and activate the innate immune system by interacting with pattern recognition receptors (PRRs), including Toll-like receptors (TLRs) and NLRPs (Nucleotide-binding oligomerization domain, Leucine rich Repeat and Pyrin domain containing, also known as NOD-like receptors). Many DAMPs are intracellular proteins derived from the nucleus or cytoplasm. Once released into the extracellular space, especially after tissue injury, they undergo changes from reducing to oxidizing environments that alter their function, associated with mitochondrial dysfunctions. Other DAMPs can originate from sources such as the extracellular matrix, mitochondria, stress granules, endoplasmic reticulum, or plasma membrane. In contrast, pathogen-associated molecular patterns (PAMPs) are conserved molecular structures found in certain microbes for the innate immunity, leading to responses such as cytokine and interferon production, helping to protect the host from infection. They are recognized by PRRs [e.g., lipopolysaccharide (LPS) from Gram-negative bacteria (recognized by TLR4), flagellin (TLR5), lipoteichoic acid and peptidoglycan from Gram-positive bacteria (TLR2), viral nucleic acids, like double-stranded RNA (TLR3) and unmethylated CpG DNA (TLR9)], triggering the innate immune response. Unlike DAMPs, which are not derived from infectious pathogens and initiate infection-related inflammation, PAMPs originate from the invasion by foreign substances and infections caused by microorganisms or viruses (Hemmi et al., 2000).

According to Deczkowska et al. (2018), neurodegeneration-associated molecular patterns (NAMPs) was proposed as endogenous danger signals commonly present in various CNS conditions, which are recognized by a battery of specific receptors constitutively expressed on microglia and trigger their transition into DAM (Figure 3, Table 1), whose primary function is to contain and remove the damage. NAMPs are released from dying or damaged neural cells, myelin debris, lipid degradation products, and extracellular protein aggregates in the context of neurodegenerative diseases and aging. This mechanism is analogous to how PAMPs and DAMPs activate immune responses in peripheral tissues (Deczkowska et al., 2018). In addition, it is noteworthy that several foundational studies have reported alterations in microglial gene expression, plausibly reflecting DAM-like phenotypes, in both aging and AD models of mice and humans (Colangelo et al., 2002; Sierra et al., 2007; El Khoury et al., 2007; Hickman et al., 2008, 2013; Table 1).

## Developmental inflammatory stress-associated glia in psychiatric disorders

### Glial heterogeneity for psychiatric disorders

Growing evidence highlights the role of glial cell heterogeneity in the pathophysiology of psychiatric disorders and suggests that, beyond neuronal dysfunction, glial cells may play critical roles in the development and progression of neurodevelopmental disorders. Schizophrenia (SCZ) is a severe and chronic psychiatric disorder that affects approximately 1% of the global population. It is characterized by complex symptoms that broadly impact social, emotional, perceptual, and cognitive functioning. Clinically, these symptoms are grouped into three major domains: positive symptoms, such as hallucinations and delusions; negative symptoms, including apathy, anhedonia, and social withdrawal; and cognitive impairments, which involve deficits in attention, working memory, and executive functions critical for goal-directed behavior (van Os and Kapur, 2009). Despite decades of research, the etiology and pathophysiological mechanisms underlying SCZ remain poorly understood.

Postmortem studies have revealed regional- and disease stage-dependent changes in microglial reactivity. The microglial density increases in the temporal and frontal lobes and region-dependent enlargement of microglial cell bodies in SCZ patients (Gober et al., 2022; see also, Snijders et al., 2021). Recent studies demonstrated that in chronic progressive SCZ, microglial reactivity and immune-oxidative pathway are elevated in early stages associated with age, accompanied by mitochondrial loss and cellular degeneration, whereas continuous schizophrenia shows consistently low microglial reactivity (Ben-Shachar, 2002; Rajasekaran et al., 2015; Laskaris et al., 2016; Weerasekera et al., 2025).

Astrocytic alterations have also been implicated in SCZ, with reports of changes in astrocytic density, morphology, and the expression of markers such as glial fibrillary acidic protein (GFAP), aquaporin-4 (AQP4), S100β, glutaminase, thrombospondin-1 (TSB-1), and excitatory amino acid transporter 2 (EAAT2; Katsel et al., 2011; Trépanier et al., 2016). Astrocytes play a key role in the development, maintenance, and function of the blood-brain barrier (BBB), and BBB dysfunction has been observed in SCZ patients (Stanca et al., 2024; Pollak et al., 2018). Neuroinflammation-driven BBB disruption may further exacerbate disease progression by allowing peripheral inflammatory mediators and immune cells to infiltrate the brain. However, findings on astrocytic changes in SCZ remain inconsistent; while some studies report reduced astrocyte numbers and marker expression, others describe increases. These discrepancies may reflect the existence of distinct glial subpopulations and heterogeneity associated with disease stage and progression.

Recent advances in proteogenomics have enabled detailed mapping of cell-type and subpopulation diversity and heterogeneity. Ling et al. (2024) identified a coordinated transcriptional program between neurons and astrocytes, termed the “synaptic neuron and astrocyte program” (SNAP), which declines in both SCZ and aging (Ling et al., 2024). This decline was observed in excitatory and inhibitory neurons as well as astrocytes, suggesting a potential link between glial dysfunction and impaired synaptic plasticity. Comprehensive analyses of neuron–glia interactions will be essential for elucidating the mechanisms underlying disorders and identifying novel therapeutic targets. Both genetic and environmental factors play critical roles in neurodevelopmental disorders. Currently, the impact of environmental stress has attracted considerable interest, based on epidemiological findings. In this review, we discuss how neurodevelopmental stress influences glial heterogeneity in the context of stress-related psychiatric disorders.

### Stress-induced glial alterations

Environmental factors, such as infections, traumatic experiences, and physical head impacts, are critical risk factors for psychiatric disorders and neurodegeneration (Giovanoli et al., 2013; Debost et al., 2017; Butler et al., 2025). These inflammatory stressors are considered to induce disease-associated glial phenotypes (Pape et al., 2019). In animal models, maternal immune activation (MIA) induced by administration of poly(I:C) or lipopolysaccharide (LPS) during pregnancy produces offspring with abnormalities in social behavior (Smith et al., 2007; Parker-Athill and Tan, 2010; Mueller et al., 2019; Hayes et al., 2022; Choi et al., 2016; Vasistha and Sawa, 2025). This phenotype resembles the symptoms observed in patients with neurodevelopmental disorders who were exposed to extrinsic stressors, such as viral or microbial infections, during critical developmental periods. Transient increases in maternal cytokines, including IL-6 and IL-17, can cross the placental barrier, leading to immune stress in the fetal brain and affecting the development and function of microglia and astrocytes (Smith et al., 2007; Yamamoto et al., 2019; Yamawaki et al., 2022; Hikosaka et al., 2022; Hayes et al., 2022; Choi et al., 2016; Vasistha and Sawa, 2025). In particular, microglia exposed to MIA exhibit blunted responsiveness to inflammatory stimuli, accompanied by epigenetic reprogramming including changes in chromatin accessibility and reduced transcription factor occupancy at open chromatin regions (Hayes et al., 2022). These glial alterations are thought to interfere with proper neural circuit formation and functions, along with chronic inflammation. In contrast, acute inflammation induces neuronal plasticity of both synaptic transmission and intrinsic excitability, leading to sickness-like behavior over a relatively short time span, indicating neural receptors against innate immunity and their distinct responses. The extent and nature of these responses depend on individual cellular, mediator, and brain region characteristics (Yamamoto et al., 2019; Yamawaki et al., 2022; Hikosaka et al., 2022).

Social defeat stress (SDS) is a model of mood disorders, induces depression-like behavior in mice (Krishnan et al., 2007; Nie et al., 2018; Kawatake-Kuno et al., 2024). Accumulating evidence indicates that single environmental factors are often insufficient for the onset of psychiatric disorders. Epidemiological studies have shown that prenatal infection combined with trauma exposure during prepuberty significantly increases SCZ risk compared to either factor alone, especially in boys (Debost et al., 2017). Animal studies further support this notion, demonstrating that combining MIA with postnatal stress produces synergistic effects on behavior and glial phenotypes (Giovanoli et al., 2013; Hikosaka et al., 2025). Hikosaka et al. (2025) reported that microglia exhibit region-specific increases and morphological alterations in response to combined MIA and repeated SDS stress (a two-hit mouse model, 2HIT). Using highly multiplexed antibody staining with imaging mass cytometry, we identified the emergence of stress-associated microglia (SAM) using a spatial proteomics method in the brains of 2HIT mice, which highly express TGFβ, APOE, MHC-class II, IL-6, and TREM2 proteins. Importantly, cerebellar Purkinje neurons exhibited reduced excitability under electrophysiology and altered action potential waveforms, implying modifications in Na^+^ and K^+^ channel expression or function. Thus, immunological alterations appear to be linked to neurophysiological function (Hikosaka et al., 2025). Microglial replacement using Ki20227, a CSF1R inhibitor, in 2HIT mice restored behaviors resembling psychiatric disorders, suggesting a causal contribution of 2HIT stress-reactive microglia, including SAM (Hikosaka et al., 2025). Such microglial transitions likely involve epigenetic reprogramming induced by MIA, coupled with metabolic adaptations in response to subsequent stress.

A population of SAM with distinct transcriptional signatures has also been reported in the hippocampus of SDS models, enriched for cytokine/chemokine signaling, cellular stress, and phagocytic activity (Goodman et al., 2024). Notably, depletion of microglia using the CSF1R antagonist PLX5622 attenuated stress-related transcriptional changes not only in microglia but also in leukocytes, endothelial cells, and astrocytes, underscoring the central role of microglia in orchestrating stress responses across multiple cell types. In a mouse SDS model of depression, Yao et al. (2025) showed that CCR5-positive neutrophils were found to infiltrate the hippocampus via the astrocyte-derived CCL5/CCR5 signaling pathway, leading to neuronal damage and depression-like behaviors. Bone marrow cell injection and CCL5/CCR5 pathway inhibition alleviated those behavioral and neuroinflammatory alterations, suggesting neutrophils and the CCL5/CCR5 axis as potential therapeutic targets for depression (Yao et al., 2025).

### Challenges and complexities in studying glial heterogeneity

The glial diversity has been delineated and is involved in anxiety, trauma-related disorders, and bipolar disorder with suicidal behavior (Myer et al., 2006; Naggan et al., 2023; Chaves-Filho et al., 2024; see also Sneeboer et al., 2019), all of which represent urgent clinical targets for intervention. Pain would be also anticipated. Importantly, interpretation of stress-related pathophysiology must extend beyond genetic background to include contextual factors such as species differences (e.g., mouse vs. human), the nature and timing of stress exposure, brain region specificity, sex, cell type, gut microbiota composition, and age. In clinical samples, the potential effects of long-term treatment with antidepressants, antipsychotics, and other mood stabilizers cannot be fully excluded. Similarly, postmortem studies face challenges in reliably documenting the history of inflammation, infections, or other chronic medical conditions. Further, the methodologies employed for RNA sequencing quantification and read mapping may contribute to an underestimation of biologically relevant signals. Specifically, transcriptional changes arising from rare cell populations or spatially localized regions may be diluted or overlooked. Conventional short-read RNA sequencing also lacks sufficient resolution to resolve full-length transcript isoforms and alternative splicing events accurately. Gene expression estimates can be biased by technical factors such as GC content, transcript length, and the wide dynamic range of expression levels. Finally, transcriptional artifacts induced by dying cells or stress responses may confound data interpretation, complicating the identification of genuine disease-associated molecular signatures.

## Disease-associated glia in neurodegenerative diseases

Chronic inflammatory responses and disease-associated glia are well known to contribute to the development of various neurodegenerative diseases. In this review, we likely focus on two neurodegenerative diseases Alzheimer's disease (AD) and amyotrophic lateral sclerosis (ALS).

### Alzheimer's disease

AD is a progressive neurodegenerative disorder characterized by the deposition of amyloid-β (Aβ) plaques and tau-associated neurofibrillary tangles, leading to synaptic dysfunction and neuronal loss (Figure 3A). Clinically, it manifests as gradual impairments in memory, cognition, language, and daily functioning, often accompanied by behavioral and emotional alterations (Alzheimer, 1907). Recent advances in genetics and epidemiology have highlighted the critical role of immune and inflammatory responses in the pathogenesis of AD. Multiple CNS cell types, including microglia, astrocytes, and vascular cells, contribute to neuroinflammation, alongside disruption of the BBB (Figure 3). Cutting-edge experimental techniques in humans and translational model mice, combined with large-scale longitudinal studies, have enabled detailed analysis of the complex interactions between the CNS and the peripheral immune system. Consequently, novel anti-inflammatory therapeutic approaches are currently being evaluated in clinical trials (Haage and De Jager, 2022; Heneka et al., 2025).

In AD, the amyloid hypothesis suggests primarily driven by the accumulation of Aβ-peptide in the brain (Hardy and Selkoe, 2002). This buildup is thought to trigger downstream effects, including the formation of tau-containing neurofibrillary tangles, due to an imbalance between Aβ production and clearance. With the advent of anti-amyloid monoclonal antibodies (mAbs), agents such as aducanumab, lecanemab, and donanemab have shown therapeutic benefits in clinical trials (Budd Haeberlein et al., 2022; van Dyck et al., 2023; Sims et al., 2023). An approximately 30% reduction in the rate of cognitive decline observed in individuals treated with these agents is considered clinically meaningful, as it reflects sustained cognitive function and a delay in progression to the more advanced stages of AD. Of note, individuals carrying the apolipoprotein E (APOE) ε4 allele—a well-known genetic risk factor for AD—are at increased risk for developing ARIA, although the underlying mechanisms remain incompletely understood. In the context of vascular pathology, it remains essential to delineate the current understanding of the relationship between AD and cerebral amyloid angiopathy (CAA; Weller et al., 1998; Yamada, 2000). CAA is classified as a form of cerebral small vessel disease and occurs in both sporadic and familial forms. Certain familial variants are characterized by prominent Aβ deposition in the leptomeninges and the walls of small-caliber and medium-sized cerebral arteries. Notably, CAA can be present independently of AD pathology in approximately 10%−20% of patients. While the prevalence of sporadic CAA increases with age, instances of early-onset CAA have been reported particularly in the context of prior neurosurgical interventions. Importantly, even in the absence of overt clinical manifestations, CAA may elicit localized inflammatory responses, potentially mediated by the recruitment of monocytes and macrophages (Yamada, 2000).

Distinct subsets of glial cells, termed disease-associated astrocyte (DAA) and disease-associated microglia (DAM), have been identified in both human patients and AD mouse models (Figures 1, 3; Keren-Shaul et al., 2017; Liddelow et al., 2017; Masuda et al., 2022). These reactive glial states and the spectrum display unique transcriptional and functional profiles, indicating roles in causing neuroinflammation, disrupting neurophysiological functions, and influencing disease progression. Aβ deposition (Spires-Jones and Hyman, 2014; Mallach et al., 2024), tauopathy (Busche and Hyman, 2020), gliosis, neural atrophy and death (De Strooper and Karran, 2016), lysosomal dysfunction (Nixon and Rubinsztein, 2024), acidification and Ca^2+^ dyshomeostasis (LaFerla, 2002), and vasculature damage and angiopathy (Zlokovic, 2011) have been investigated long time. High-throughput techniques such as RNA sequencing and mass spectrometry-based proteomics have been instrumental in uncovering these molecular signatures (Table 2). Still, notable limitations exist when extrapolating findings from mouse models to human AD pathology. Additionally, glial behavior appears to be modulated by biological variables such as age and sex. Integrated multi-omics approaches, particularly combining transcriptomic and proteomic technologies, have gained to identify cell-type-specific and gene and proteomic signatures across various stages of AD (Mathys et al., 2019; Zhou et al., 2020; Hu et al., 2021; Lin L. et al., 2023; Mallach et al., 2024; Wu et al., 2024; Wang et al., 2024; Johnston et al., 2025; Humphrey et al., 2025).

#### DAM in AD

DAM is a unique subtype of microglia that emerges early during AD progression and is characterized by its localization near Aβ plaques, along with phagocytic and lipid-metabolizing features (Figure 3, Table 1; Keren-Shaul et al., 2017; Deczkowska et al., 2018; Mallach et al., 2024). DAM exhibit a dynamic two-stage activation sequence: an early TREM2-independent phase followed by a TREM2-dependent phase. NAMPs play a role in the transition from homeostatic to Stage 1 DAM and Stage 2 DAM via Trem2 signaling (Deczkowska et al., 2018). The first stage is Trem2-independent and involves a reduction in homeostatic microglia checkpoint genes such as *Cx3cr1, P2ry12*, and *P2ry13*, along with upregulation of genes including the Trem2-signaling adaptor *Tyrobp, Apoe*, and *B2m*. The second stage is Trem2-dependent and is marked by increased expression of genes involves in lipid metabolism and phagocytic pathway genes (e.g., *Lpl, Cst*, and *Cd9*), causing excessive inflammatory responses and neurodegeneration (Keren-Shaul et al., 2017; Deczkowska et al., 2018). Supporting this model, transcriptomic analysis of 3xTg-AD mice at different disease stages (2, 10, and 20 months) revealed age-related increases in DAM-associated genes such as *Trem2, Tyrobp, Clec7a*, and *Cd68*, particularly in aged AD mice compared to young AD mice and aged-wild-type controls (Lambracht-Washington et al., 2023). Correspondingly, they found that plaque pathology progressed with age, from no plaques at 2 months to sparse plaques in the subiculum at 10 months, and abundant plaques in hippocampus subiculum at 20 months old 3xTd-AD mice (Lambracht-Washington et al., 2023). An APOE-dependent molecular signature is commonly observed in AD-associated microglia. Targeting the Trem2-APOE pathway can restore the homeostatic signature of microglia in ALS and AD mouse models, potentially preventing neuronal loss and promote tolerogenic T-cell response (Krasemann et al., 2017). Additionally, the APOE-ε4 allele is the strongest genetic risk factor for both early- and late-onset AD, contributing to increased amyloid deposition and disease progression (Kim et al., 2009).

As reported by Rangaraju et al. (2018), DAM can be transcriptionally separated into pro-inflammatory and anti-inflammatory subclusters, implying the existence of functionally distinct states. This duality or multiple states suggest that DAM may exert context-dependent cell transition under different stress conditions, acting as a double-edged sword. Indeed, several studies have proposed that DAM contribute to the clearance of neuronal debris, including synaptic structure, a function inferred from their transcriptional profile as described by Keren-Shaul et al. (2017). For instance, TREM2 is a key receptor regulating microglial function and a marker protein of DAM phenotype, and its deficiency impairs the ability of microglia to respond effectively to myelin damage, thereby promoting demyelination and neurodegeneration (Poliani et al., 2015). Lipids exposed by damaged myelin have been identified as activators of TREM2, underscoring its role in microglial responses to white matter injury (Poliani et al., 2015). In contrast, microglia in close proximity to amyloid plaques (fluorescent Congo-red derivative, methoxy-XO4 positive, XO4^+^) and those distant from plaques (XO4^−^) have been isolated and analyzed from AD model mice. XO4^+^ microglia exhibited dysregulated expression of AD-associated genes, while XO4^−^ microglia showed transcriptional signatures resembling accelerated aging and reduced phagocytic capacity. The study also identified hypoxia inducible factor-1α (HIF-1α) as a potential regulator of synaptosome phagocytosis (Grubman et al., 2021). As neuritic plaques form progressively around Aβ deposits in AD, lysosomal dysfunction emerges early in dystrophic neurites. Proteins such as saposin C and LAMP1 accumulate abnormally, while lysosomal hydrolases are notably absent, suggesting that lysosomal processing is already compromised at early plaque stages. As plaques grow, lysosomal signals shift from neurons to DAM, with a corresponding reduction in lysosomal proteins within dystrophic neurites. The study proposed that early lysosomal failure within dystrophic neurites promotes continued amyloid plaque accumulation and microglia recruitment in AD (Sharoar et al., 2021). The increasing Aβ aggregation also led to the release of nitric oxide, reactive oxygen species (ROS), and proinflammatory cytokines which are factors that may contribute to neuronal death (Akiyama et al., 2000).

#### APOE4 difference from other APOE isoforms

Apolipoprotein E (APOE) is a protein that transports cholesterol and lipids. In the brain, APOE is mainly produced by astrocytes and secreted as HDL-like particles. APOE is involved in lipid transport into cells through the LDL receptor (LDLR). The apolipoprotein E ε4 (APOE4) allele confers the highest risk for AD, whereas APOE2 acts protectively. APOE4 differs significantly from the other two major isoforms, APOE2 and APOE3, in both structure and function. There are three main genetic variants (isoforms) of APOE, distinguished by specific amino acid substitutions. APOE4 has arginine at amino acid position 112, whereas APOE3 has cysteine. This substitution promotes an abnormal salt bridge between the N-terminal and C-terminal domains, causing structural changes and altering the protein's folding and conformation. The structural alteration in APOE4 impairs its ability to bind and transport lipids (cholesterol, phospholipids) efficiently, compared to APOE3 and APOE2. APOE4 is less stable and more prone to proteolytic degradation, leading to reduced functional persistence inside and outside the cell. Interactions with receptors such as LDLR and LDL receptor-related protein 1 (LRP1) are pivotal for APOE-mediated lipid and Aβ metabolism. Of note, APOE4 exacerbates mitochondrial dysfunction and oxidative stress within neurons, thereby amplifying neurotoxicity and contributing to neurodegenerative processes (Chen et al., 2021).

#### TREM2 molecules in AD

Heterozygous rare variants in TREM2 (p.R47H) significantly increase in the risk of AD. Given the reported anti-inflammatory effect of TREM2 in the brain, the R47H substitution may lead to an increased predisposition to AD (Jonsson et al., 2013; Guerreiro et al., 2013; Slattery et al., 2014). One of the characteristics of DAM is the expression of TREM2, which marks a distinct population of microglia and macrophages. However, the origin of these cells remains unclear; it is not yet clear whether they are derived from brain-resident microglia/macrophages, from peripheral tissues that have migrated into the brain, or from cells residing at the vascular interface. Further investigation into their origins is warranted. In recent years, TREM molecules have attracted increasing attention in the field of neurodegenerative diseases. An agonistic monoclonal anti-body (mAb) of TREM2, AL002, was shown to induce microglia proliferation and reduces pathology in AD mice (Wang S. et al., 2020). TREM2 had been highlighted for its potential therapeutic role, with activating antibodies and small-molecule drugs showing promise for the treatment of dementia. At the time of writing, phase II clinical trials are underway for some of these agents with negative results in INVOKE-2 trial (van Lengerich et al., 2023; Colonna and Holtzman, 2025). In contrast, inhibition of TREM1 has also demonstrated protective effects in mouse models of neurodegenerative diseases (Wilson et al., 2024). We believe that targeting TREM2 in neurodegenerative diseases holds significant therapeutic promise. However, its successful application will require further elucidation of TREM biology in humans, regarding the cell-type specificity of TREM expression (e.g., in astrocytes, oligodendrocytes, and neurons), species differences between humans and mice, immature metabolic and mitochondrial features of *in vitro* culture (e.g., BV and iPSC-derived cells), early-onset or late-onset, and the temporal dynamics of disease progression with senescence. In addition, studies often use human samples labeled as pathologically normal, which may seem unrelated but can still affect analytical outcomes. These aspects are beyond the scope of the present study and have been discussed in detail in recent reviews (Colonna, 2023; Colonna and Holtzman, 2025).

#### DAA and reactive astrocytes in AD

Alongside microglia, astrocyte heterogeneity plays a crucial role in AD pathogenesis (Figure 3). Astrocytes exhibit significant morphological, molecular, and functional changes in response to CNS pathologies such as neurotrauma, stroke, brain hemorrhage, infections, epilepsy, and AD (Escartin et al., 2021). A unique reactive astrocyte specific to AD, termed DAA when transcriptomically defined (Liddelow et al., 2017), has been positioned near Aβ-plaque and DAM (Figure 3B; Mallach et al., 2024). These DAAs appear early in the disease, in both sexes, primarily in the cortex and hippocampus (Habib et al., 2020).

While astrocytes were historically considered to appear morphological changes in response to injury, trauma, aging, and neurodegeneration (Stephenson et al., 1999; Myer et al., 2006; Furman et al., 2012; Anderson et al., 2014; Crowe et al., 2016; Sirko et al., 2015; Itoh et al., 2018), it is now understood that they also display inherent functional heterogeneity (Liddelow et al., 2017; Patani et al., 2023). This shift in perspective followed comprehensive single-cell transcriptomic studies, which revealed that astrocytes differ in function and gene expression, depending on the specific environmental stressors and pathological conditions (Liddelow et al., 2017; Boisvert et al., 2018; Clarke et al., 2018; Endo et al., 2022; Patani et al., 2023; Qian et al., 2023; Sheehan et al., 2023; Ahmed et al., 2024; Kim et al., 2024; Yu et al., 2024). Smith et al. (2020) demonstrated that activation of the unfolded protein response, specifically through phosphorylation of PERK (protein kinase R-like ER kinase), induces a unique reactive state in astrocytes. This state altered their secretome and impaired their synaptogenic function *in vitro*. *In vivo* experiments of prion-disease model mice showed cytotoxicity of PERK-dependent reactive astrocytes against neurons. Selective inhibition in astrocytes alone was sufficient to prevent non-cell-autonomous neuronal loss and prolong survival, as a mechanism of neurodegeneration driven by reactive astrocytes (Smith et al., 2020). Meanwhile, still marker protein expressions are under arguments in the context of potential inconsistency between transcriptome and protein expression, with their functionality (Table 2).

Here, we aim to introduce the potential involvement of reactive astrocytes in AD pathology, characterized by the expression of distinct markers such as GFAP, TSPO, and MAO-B. Understanding these markers may help readers appreciate their advantages in non-invasive diagnostic applications.

##### GFAP

Astrocytes are known to be classified into two subpopulations, depending on GFAP expression. For instance, DAAs express high levels of GFAP, a common marker of astrocyte activation (Liddelow et al., 2017). Habib et al. (2020) comprehensively provided corroborating evidence for reactive astrocytes or DAAs located close to Aβ plaques (Habib et al., 2020). A following study identified the specific astrocyte subpopulation (GFAP^low^, AQP4^+^, CD63^+^), verified in both humans and mouse AD models, enriched in early AD and diminished in later stages (Wei et al., 2024). However, pathophysiological features of AD such as neuronal loss, gliosis, and neurofibrillary tangles of phosphorylated tau have not been thoroughly investigated. If gliosis has progressed, the expression of GFAP near the affected area would be expected to decrease or there are regional differences. As experimental evidence, GFAP expression is markedly upregulated in reactive astrocytes during CNS inflammation, a characteristic feature of gliosis observed in AD, resulting in a 1.2–3.3-fold increase in GFAP levels (Kamphuis et al., 2014). GFAP expression is particularly evident in astrocytes surrounding amyloid plaques. Under physiological conditions, GFAP is almost undetectable in the blood. However, CNS inflammation can cause GFAP to leak into the extracellular space and enter the systemic circulation. Elevated serum GFAP has been proposed as a peripheral biomarker of CNS pathology (Fukuyama et al., 2001; Edwards and Robinson, 2006).

##### TSPO

The translocator protein (18 kDa; TSPO) is a mitochondrial protein located on the outer membrane, which has brought attention as a neuroinflammatory biomarker. TSPO is an important regulator of stress responses (ROS and cell death) and mitochondrial dysfunction involved in neurodegenerative diseases, as well as mental and stress-related disorders such as autism spectrum disorder, bipolar disorder, and depression. Activation of TSPO promotes the production of endogenous neurosteroids, as in steroid synthesis and mitochondrial function. TSPO ligands have been used as markers of neuroinflammation and microglial activity in positron emission tomography (PET) imaging. Endogenous ligands of TSPO, cholesterol and porphyrins, have been reported to regulate neuroplasticity and exhibit antidepressant and anxiolytic effects in animals and humans (Lejri et al., 2019; Rupprecht et al., 2022).

Due to the correlation between biological findings, cellular damage, aging, and AD pathology, TSPO-PET remains a widely used (Repalli, 2014). TSPO is known as the reactive microglia marker; however, it is also expressed in reactive astrocytes, endothelial cells, and vascular smooth muscle cells in AD brains (Barron et al., 2013; Gui et al., 2020). TSPO expression is dependent on disease stage and species differences because of the biological feature *in vivo* (Tournier et al., 2020). While biological evidence as the marker in AD is still ambiguous, future studies should clarify what changes in TSPO-PET truly represent to improve its diagnostic and research accuracy (Gouilly et al., 2022; Appleton et al., 2025).

##### MAO-B

MAO-B (Monoamine Oxidase B) is highly and selectively expressed in astrocytes, and its upregulation in reactive states exhibits greater astrocyte specificity than that of TSPO. It catalyzes the oxidative deamination of monoamines such as dopamine, thereby modulating neurotransmitter levels (Petrelli et al., 2020). Petrelli et al. (2020) provided compelling evidence that astrocytes regulate dopamine homeostasis in the developing prefrontal cortex, crucial for cognitive circuit formation. Importantly, MAO-B activity increases with age and under neuroinflammatory or neurodegenerative conditions (Oreland and Gottfries, 1986; Ekblom et al., 1993; Fowler et al., 1997). MAO-B serves as a target for PET imaging using Carbon-11 and Fluorine-18 radioligands, such as [^11^C]-L-deprenyl and [^18^F]SMBT-1, respectively, which have been translated into clinical applications (Rodriguez-Vieitez et al., 2016; Arakawa et al., 2017; Harada et al., 2021; Koshimori et al., 2023). The high astrocyte specificity of MAO-B around dense-core Aβ plaques enables more precise interrogation of astrocyte-related pathophysiology in the living brain in the context of neurodegenerative disease (Jaisa-Aad et al., 2024). The utility of these tracers is influenced by their physical half-life, metabolic stability, and whether they exhibit reversible or irreversible binding kinetics.

#### BBB dysfunction in Alzheimer's disease

The BBB is composed of endothelial cells, pericytes, capillary basement membrane, and astrocyte end-feet (Cabezas et al., 2014; Segawa et al., 2021). BBB breakdown is well studied in aging human living brains and post-mortem tissues in neurodegenerative disorders (Montagne et al., 2015; Sweeney et al., 2018). Recent imaging studies reveal that BBB breakdown and glymphatic system impairment associated with gliosis occur early in aging, particularly in the hippocampus, and are exacerbated in mild cognitive impairment, correlating with pericyte injury (Montagne et al., 2015). This early BBB dysfunction and glymphatic system impairment may contribute to the initiation of neurodegenerative processes such as those seen in Alzheimer's disease (Sweeney et al., 2018).

Magnetic resonance imaging (MRI)-guided low-intensity focused ultrasound (“FUS”) has emerged as a safe, noninvasive strategy to transiently and repeatedly open BBB in targeted brain regions, enabling localized drug delivery and facilitating Aβ clearance. In the largest and longest follow-up study to date (up to 12 months), patients with mild AD underwent FUS targeting the hippocampus, frontal, and parietal lobes, with no serious adverse events, full BBB closure within 48 h, and cognitive outcomes comparable to those seen with natural disease progression (Rezai et al., 2022). Notably, PET imaging revealed region-specific reductions in amyloid burden, and a separate study combining FUS with anti-amyloid antibody administration (specifically, aducanumab infusions) suggested enhanced accessibility of anti-amyloid antibody in parenchyma and plaque clearance in sonicated regions. These findings support the safety and potential disease-modifying effects of FUS-mediated BBB opening in early-stage AD, although larger trials are warranted to establish clinical efficacy (Rezai et al., 2024).

#### Sex-dependent glial effects in AD

Sex differences in glial reactivity contribute to the progression of AD. Female brains exhibit a stronger correlation between phosphorylated tau (p-tau) and neurodegeneration compared to males (Vila-Castelar et al., 2025; Biel et al., 2025). This effect is further exacerbated by the APOE-ε4 and sTREM2 pathways (Biel et al., 2025), which likely underlie the higher prevalence and rapid progression of AD in woman (Vila-Castelar et al., 2025). Sex difference also influences microglial density and soma size in a brain region and age-dependent manner. In the hippocampus and cortex of 3- and 13-week-old mice, males displayed larger somas at 13 weeks and increased expression of MHC class I and MHC class II molecules in these regions (Guneykaya et al., 2018). Furthermore, in the basic membrane property, male microglia exhibited greater membrane currents and stronger ATP responses via P2X receptors, consistent with proteomic data showing elevated expression of P2X4, P2X7, and P2Y12 receptors (Guneykaya et al., 2018). Considering that the human APOE isoform further regulates Aβ clearance efficiency, and that sex and age modulate APOE-related AD risk via hormonal and aging factors (Cacciaglia et al., 2022), APOE-ε4 is associated with more severe cognitive decline and increased AD risk, particularly in individuals aged 70–80 (Cacciaglia et al., 2022). Estrogen may interact with APOE, and its decline during aging in women exacerbates APOE-ε4-related AD phenotypes (Cacciaglia et al., 2022; Valencia-Olvera et al., 2023). These findings highlight the interplay between hormonal and genetic factors in shaping sex-specific trajectories of AD onset and progression.

### Amyotrophic lateral sclerosis

#### Glial contributions to ALS progression

Amyotrophic lateral sclerosis (ALS) is a devastating neurodegenerative disease characterized by the progressive loss of motor neurons (Charcot, 1874). Akin to other neurodegenerative disorders, inflammation in the affected regions, such as the motor cortex and spinal cord, is driven by a complex interplay between genetic predispositions and environmental influences. More than 40 genetic mutations have been reported implicated in ALS pathophysiology, such as *C9orf72* (chromosome 9 open reading frame 72), *SOD1* (superoxide dismutase 1), *TARDBP* (TAR DNA-binding protein 43, coding for TDP43), and *FUS* (fused in sarcoma; Goutman et al., 2022). Though many genetic risk factors have been identified in familial ALS (fALS) cases, the majority cases of ALS are sporadic and have no clear genetic cause.

The intronic G_4_C_2_ hexanucleotide repeat expansion in the *C9orf72* gene is the most common genetic cause of ALS, present in both fALS and sporadic ALS (sALS) cases. It is strongly associated with both frontotemporal dementia (FTD), either alone or as part of ALS-FTD. *C9orf72* repeat expansion causes cellular dysfunction through dipeptide repeat production and C9orf72 loss-of-function. Recent studies have shown that *C9orf72* mutations alter the immune reaction. C9orf72 protein can be recognized by T cells as an autoantigen, and this ALS-associated T cell autoreactivity was found broadly in ALS patients, though it was particularly high in *C9orf72* mutation carriers (Michaelis et al., 2025). Single-nuclei transcriptomes from sALS and *C9orf72* ALS revealed that *C9orf72* hexanucleotide repeat expansion impairs the microglial cell state transition to activated state compared to sALS (Masrori et al., 2025). They also found that astrocyte responsiveness was diminished in *C9orf72* mutants.

*SOD1* mutation is another common genetic factor, which is associated in around 20% cases of fALS. Mutations in *SOD1* increase protein misfolding and lead to the production of insoluble SOD1 aggregates in cytoplasm. Wild type SOD1 can be misfolded by the aberrant translation, post-translational modification or oxidative modifications, due to the pathological conditions of the CNS environment, and produce toxic insoluble aggregates, as mutated SOD1 (Bosco et al., 2010; Durazo et al., 2009). Misfolded wild type SOD1 is observed in sALS cases and the existing treatments for SOD1-related fALS, such as the antisense oligonucleotide therapy Tofersen, may have a possibility to be applied to these sALS cases (Marlow et al., 2025). In mouse model, Frakes et al. (2014) showed that microglial NF-κB is involved in the disease-like phenotypes progression in SOD1-G93A mice and suppression of NF-κB reduces proinflammatory microglial activation (Frakes et al., 2014).

A distinctive pathological feature of ALS is the mislocalization and aggregation of phosphorylated TDP-43 (pTDP-43), a nuclear RNA-binding protein encoded by *TARDBP*. Phosphorylation of TDP-43 disrupts its nuclear localization, leading to cytoplasmic aggregation (Ayala et al., 2008; Hasegawa et al., 2008), which has been observed in over 95% of ALS cases (Neumann et al., 2006). A recent study revealed that TDP-43 accumulation within stress granules, together with oxidative stress, collectively induces intra-condensate demixing, leading to a TDP-43-enriched phase that forms pathological cytoplasmic aggregates (Yan et al., 2025). Mislocalized TDP-43 in neurons and glial cells disrupts RNA processing and proteostasis, leading to cellular dysfunction that contributes to pathological glial alterations and neurodegeneration. Therefore, elucidating the molecular mechanisms underlying glial contributions to ALS pathogenesis is crucial for advancing our knowledge of the disease and the development of targeted therapeutic strategies.

#### DAM in ALS

DAM has been identified in ALS patients and mouse models, as AD (Keren-Shaul et al., 2017; Jauregui et al., 2023; Zelic et al., 2025). The elevated levels of microglia activating factors, including M-CSF, MCP-1/CCL2, TNF-α, IL6, INF-γ, and TGF-β, were identified in the CNS of ALS patients or ALS mouse and cellular models (Yoshihara et al., 2002; Hensley et al., 2003; Henkel et al., 2004; Sargsyan et al., 2005). Elevated *TREM2, MS4A, CD33, APOE*, and *TYROBP* gene expression were also observed in postmortem ALS spinal cord samples (Jauregui et al., 2023). Though DAM has been detected across multiple neurological disease models, the composition of disease related microglia and macrophage clusters differs between disease—phagocytic subtype predominant in AD, while inflammatory subtype predominant in ALS (Wishart et al., 2023). Recent findings emphasize that activated state microglia is not a homogenous cell population. According to Tuddenham et al. (2025), microglia in the human ALS motor cortex and spinal cord were classified into around seven subtypes. The predominant subset was undifferentiated phenotype with dysregulated respiratory electron transport, suggesting that disruptions in both metabolism and mitochondrial function are involved. The subset with DAM phenotype was significantly depleted (or reduced) in human ALS, suggesting that loss of DAM might be a feature of late disease stage.

In ALS, cytosolic TDP-43 elicits innate immune responses through mitochondrial damage and activation of the cGAS/STING pathway (Ma et al., 2024; Yu et al., 2020), thereby promoting inflammasome production and driving microglial activation toward DAM-like phenotypes (Quek et al., 2022; Heneka et al., 2014). TDP-43-induced inflammatory responses in microglia were attenuated when treated with an IL-6 trans-signaling inhibitor *in vitro*, suggesting that IL-6 trans-signaling may act as a potential driver of inflammation in ALS pathogenesis (Risby-Jones et al., 2025). DAM release superoxide radicals (i.e., ROS), nitric oxides, and pro-inflammatory cytokines and factors (IL-1α, TNF-α, C1q), which induce neurotoxic C3^+^ reactive astrocytes (Liddelow et al., 2017). On the other hand, DAM can also exert neuroprotective functions through TREM2 signaling, as evidenced by the finding that TREM2 deficiency exacerbates pathological TDP-43 inclusions, motor dysfunction, and neurodegeneration (Xie et al., 2022). Studies from mouse models have revealed that the transcriptional profile of the rod-shaped microglia was akin to that of DAM (Matsuba et al., 2025) and that rod-shaped microglia interact with neuronal dendrites, attenuate motor cortical hyperactivity during an early stage of TDP-43-associated neurodegeneration (Xie et al., 2025). TREM2 deficiency in TDP-43 mouse model leads to a marked reduction in rod-shaped microglia, accompanied by increased neuronal activity (Xie et al., 2025). These findings suggest that rod-shaped microglia play a neuroprotective role in the early phase of neurodegeneration. Whether DAM are neuroprotective or neurotoxic likely depends on the disease context and state, warranting further investigation.

#### DAA in ALS

Transcriptome analyses have identified distinct DAAs in postmortem samples from both sporadic and familial ALS cases (Pineda et al., 2024). According to Fioretti et al. (2025), astrocytes gene signatures are upregulated during the early symptomatic stage, characterized by proliferation, and subsequently they decline as pro-inflammatory genes become upregulated during disease progression in TDP-43 mouse model.

DAA, particularly neurotoxic C3^+^ reactive astrocytes, can be induced by factors such as IL-1α, TNF-α, and C1q, released from activated neuroinflammatory microglia, as mentioned (Liddelow et al., 2017). These astrocytes exhibit reduced glutamate reuptake and diminished production of neurotrophic factors, ultimately leading to motor neuron loss. Crosstalk between microglia and astrocytes further contributes to neurodegeneration, as demonstrated by prolonged survival in SOD1 mouse model when this intracellular communication was blocked (Guttenplan et al., 2020b). Moreover, affected neurons themselves can influence astrocyte states. MicroRNA-218 (miR-218) released from dying motor neurons in ALS can directly modify astrocytes into disease-associated phenotypes (Hoye et al., 2018). Extracellular miR-218 is internalized by astrocytes, leading to downregulation of the glutamate transporter EAAT2 (also called GLT-1), and inhibition of miR-218 using antisense oligonucleotides (ASO) in an ALS mouse model rescued EAAT2 expression and astrocyte function (Hoye et al., 2018).

Astrocyte reactivity contributes directly to neurodegeneration. Bone morphogenetic protein 4 (BMP4), which promotes astrocytogenesis and its activation, and its downstream signaling were revealed to play a key role in astrocytosis (Shijo et al., 2018). BMP4 was up-regulated in reactive astrocytes of SOD1 ALS rat model spinal ventral horns, and BMP4 knockdown through ASO suppressed glial activation and ameliorated the motor dysfunction (Shijo et al., 2018). Additionally, ALS patient-derived induced astrocytes (iAstrocytes) were neurotoxic toward mouse motor neurons and their expression levels of disease-associated markers, including GFAP, CX43 and Ki-67, and miRNA profiles varied among patients, suggesting the astrocytes heterogeneity in ALS (Gomes et al., 2022). Recent study shows that overactivation of MYC detected in ALS astrocytes induce alterations in EV release and these alterations trigger astrocyte-to-neuron miscommunication, resulting in reduced support of neighboring neurons (Fioretti et al., 2025).

#### Therapeutic approaches targeting glial cells

Given the prominent role of glial cells in the onset and progression of ALS, modulating their activity is one of the promising therapeutic strategies in ALS. As described above, inhibition of IL-6 trans-signaling has been shown to mitigate inflammation responses (Risby-Jones et al., 2025), and targeting miRNAs has a potential to modulate glial cell functions (Hoye et al., 2018; Gomes et al., 2022).

Receptor-interacting protein kinase1 (RIPK1) is a central regulator of cell death pathways, including apoptosis, necroptosis, and inflammation (Ofengeim et al., 2017). RIPK1 activity is elevated in ALS and has been proposed as a key mediator of glial state transitions in ALS pathogenesis (Mifflin et al., 2021; Wei J. et al., 2023; Zelic et al., 2025). A distinct subset of microglia, exhibiting pro-inflammatory gene signatures has been implicated in ALS, with RIPK1 expression markedly upregulated in SOD1 mouse models. Inhibition of RIPK1 was sufficient to suppress microglial activation (Mifflin et al., 2021). Moreover, single-nucleus RNA sequencing of postmortem ALS spinal cords identified glial populations enriched for inflammatory and activation-associated markers, many of which converge on RIPK1 signaling and necroptotic cell death pathways (Zelic et al., 2025). In human tri-culture systems comprising induced pluripotent stem cell (iPSC)-derived motor neurons, astrocytes, and microglia, RIPK1 activation modulated cytokine profiles, several of which mirrored increases or decreases in the cerebrospinal fluid (CSF) of ALS patients (Zelic et al., 2025). Consistently, Wei J. et al. (2023) demonstrated that both RIPK1 and IL-8 were elevated in the serum of ALS patients, with RIPK1 concentrations correlating with symptom severity. Administration of primidone, an FDA-approved drug as a RIPK1 inhibitor, significant delayed disease onset and improved motor performance in SOD1 mouse models. Notably, primidone reduced serum RIPK1 levels in ALS patients (Wei J. et al., 2023). Multiple clinical trials are currently evaluating additional RIPK1 inhibitors for ALS therapy.

There is another treatment approach aimed to modulate inflammation. Allison and Ebert (2024) demonstrated that combination of interleukin 10 (IL-10) increase and CCL2/MCP-1 signaling blockade with CCL2 neutralizing antibodies (CCL2nAb) specifically targeting astrocytes attenuated glial activation, using iPSCs-derived cultures from ALS patients harboring *SOD1* and *C9orf72* mutations. CCL2/MCP-1 is known to be secreted by monocytes and reactive microglia (Sargsyan et al., 2005). Although the underlying mechanisms were different between *SOD1* and *C9orf72* models, treatment of cultured astrocytes with IL10/CCL2nAb was effective (Allison and Ebert, 2024). A monoclonal antibody, ACI-6677, was developed by AC Immune SA to target the protease-resistant amyloid core of pathological TDP-43. ACI-6677 inhibits aggregation, promotes phagocytic clearance, and prevents the intercellular spread of TDP-43 pathology in murine models (Chevalier et al., 2024). Additionally, a novel vectorized monoclonal antibody (vmAb), ACI-5891, was developed to target pathological TDP-43 in ALS and FTD (Val et al., 2025). Delivered via AAV9, a single intracisternal dose enabled sustained brain expression for months and reduced pathological TDP-43 levels in translational mice (Val et al., 2025). In antisense oligonucleotide (ASO) therapies, a *C9orf72* sense transcript-targeting antisense oligonucleotide BIIB078 exhibited robust efficacy in the preclinical mouse and cellular models (Donnelly et al., 2013; Jiang et al., 2016); however, in phase 1 clinical trial, BIIB078 failed to exert meaningful effects on CNS pathologies and had no significant impact on neurofilament levels but paradoxically increased inflammatory marker CCL26, and its development has been discontinued (van den Berg et al., 2024; McEachin et al., 2025). Thus, these findings underscore the notion that translational mouse models replicate only a subset of human pathology, often readily show therapeutic effects, and lack the complexity of aged human tissues.

Although these therapeutic strategies are still in development, they have the potential to suppress ALS progression by directly targeting specified glial cell populations, applying Treg, and suppressing age-associated chronic inflammation (Gendron and Petrucelli, 2023).

## Glial innate immunity in neurodegenerative diseases

Early studies established that cytosolic DNA can elicit innate immune responses and innate inflammation, inducing type I interferons (IFNs), yet the molecular mechanism underlying this sensing remained unresolved (Seth et al., 2006). The breakthrough came when Chen and colleagues (Sun et al., 2013) discovered that cytosolic DNA triggers the synthesis of a cyclic dinucleotide, cyclic GMP-AMP (cGAMP). This molecule was subsequently shown to bind and activate stimulator of interferon genes (STING), a known adaptor protein that mediates downstream immune signaling. Subsequent efforts to identify the enzyme responsible for cGAMP production led to the characterization of cyclic GMP-AMP synthase (cGAS), a cytosolic DNA sensor that directly binds double-stranded DNA and catalyzes cGAMP synthesis upon activation (Sun et al., 2013).

### cGAS/STING pathway in microglial diseases

In the context of neurodegeneration, amplification of microglial cGAS-STING signaling, along with IFN production and cGAS-driven senescence, has been implicated as a disease-promoting mechanism in female mice (Carling et al., 2024). Notably, cGAS- and BAX-dependent microglial senescence was exacerbated by the combined presence of the APOE4 allele and the TREM2^R47H^ variant, indicating both associated with increased risk and earlier onset of Alzheimer's disease and their potential role as upstream modulators of AD pathogenesis (Carling et al., 2024). In ALS, cytosolic TDP-43 translocated into mitochondria via TOM20, triggering ROS production and mitochondrial DNA (mtDNA) release through the mitochondrial permeability transition pore (mPTP). The released mtDNA was subsequently recognized by cytosolic cGAS, activating the STING pathway and inducing a pro-inflammatory response by IFN, TNF, IL-6, and IL-1β (Yu et al., 2020).

With regards to cellular senescence, Yang et al. (2017) demonstrated that mouse embryonic fibroblasts (MEFs) derived from *cGas*^−/−^ mice exhibited reduced senescence and underwent more rapid spontaneous immortalization, compared to wild-type counterparts. Loss of cGAS abolished senescence phenotypes and senescence-associated β-galactosidase (SA-β-Gal) activity, suggesting that cGAS is essential for cellular senescence (Yang et al., 2017). In senescent cells, chromatin fragments leak into the cytoplasm due to DNA damage response (DDR) and impaired nuclear envelope function. It is noteworthy that Glück et al. (2017) revealed that innate DNA sensing through cGAS governs the senescence program and the senescence-associated secretory phenotypes by detecting cytosolic chromatin fragments in senescent cells. In response to oxidative stress, cGAS activation via STING drives the expression of senescence-associated secretory phenotype (SASP) factors, including TNF-α, IL-6, CXCL2, CXCL10, CCL3, and CCL5. SASP is the set of inflammatory signals and proteins that senescent cells release to influence nearby cells and tissues; thereby cGAS-STING promotes paracrine senescence (Glück et al., 2017).

### cGAS-STING signaling in astrocytes and CNS inflammation

In astrocytes, AD model mice (5xFAD) crossed with the *Cgas*^−/−^ line exhibited a reduction in GFAP^+^ cell area, indicating the involvement of the cGAS-STING pathway (Xie et al., 2023). The transcriptional repressor Yin Yang 1 (YY1) has also been implicated in cGAS signaling (Jiang et al., 2023). cGAS expression was pronounced not only in senescent microglia but also in astrocytes. Knockdown of the cGAS-STING pathway prevented astrocyte senescence both *in vitro* and *in vivo*, thereby ameliorating Parkinson's disease-like pathology in MPTP-treated mice, a model that exhibits persistent Parkinsonian symptoms. Mechanistically, STING was shown to directly interact with YY1, inhibiting its nuclear translocation and consequently promoting transcription of lipocalin-2 (LCN2). These findings suggest that the cGAS-STING-LCN2 axis contributes to age-associated neurodegeneration (Jiang et al., 2023). In humans and mice, cGAS has a negative regulatory role in homologous recombination repair. Surprisingly, however, in the naked mole-rat, four specific amino acid substitutions in cGAS promote DNA repair and confer a greater capacity to stabilize the genome, counteract cellular senescence and organ aging, and to enhance longevity and health span via evolution (Chen et al., 2025).

As described, the cGAS-STING pathway is a canonical in innate inflammatory stress responses, not only within neurons and glial cells of the CNS but also across a broad range of peripheral tissues, contributing to diverse pathological contexts such as cancer (Chakraborty et al., 2024; Guo et al., 2025), SARS-CoV-2 infection (Li M. et al., 2021), ALS (Yu et al., 2020), multiple sclerosis (Woo et al., 2024), and neovascular age-related macular degeneration (Tanaka et al., 2024). Emerging STING antagonists, including STING-IN-2 (C-170) and SN-011, hold promise as alternative therapeutic agents with potentially improved efficacy and tolerability (Liu et al., 2021).

## Reconsideration of the heterogeneity of reactive glia in neurodegenerative diseases

### DAM heterogeneity and different roles

In this section, we selected several recent studies addressing the issues discussed above. First, SPI1 (also known as PU.1) is a transcription factor crucial for microglia viability and differentiation, located within a genome-wide significant AD-risk locus, where reduced expression is associated with delayed AD onset. Ralvenius et al. (2023) analyzed single-cell transcriptomic data from microglia in human AD brains and found PU.1-binding motifs enriched among differentially expressed genes (DEGs). In hippocampal tissues of neurodegenerative transgenic mice, genomic PU.1 binding occupancy was vastly increased. Through a targeted chemical screen, the authors identified A11, a small molecule with anti-inflammatory properties, which appears to regulate gene expression putatively by recruiting a repressive complex containing MECP2, HDAC1, SIN3A, and DNMT3A to PU.1 bound regions. In mouse models, A11 treatment ameliorated neuroinflammation, preserved neuronal integrity, mitigated AD pathology, and improved cognitive performance (Ralvenius et al., 2023).

An inducible Clec7a-CreERT2 mouse line enables selective genetic tracing of proliferative-region-associated microglia (PAM) and DAM in the CNS (Barclay et al., 2024). Single-cell RNA sequencing of labeled microglia revealed both convergent and divergent transcriptional programs across developmental and disease contexts. Long-term *in vivo* tracing demonstrated state transitions, exhibiting reversible microglial plasticity. State-specific ablation during demyelination established that DAM exert protective functions for effective remyelination in a multiple sclerosis model. However, it is prudent to consider that CLEC7A is not a specific marker reflecting all DAM features. Furthermore, it remains uncertain whether the signature gene expression pattern observed in mouse models truly represents human pathology when comparing expression patterns in human patient samples, and how reversible trained DAM memories are. In aging, white-matter-associated microglia (WAMs) also show DAM-like properties. The Clec7a-CreERT2 driver also labels CNS border-associated macrophages (BAMs) and certain peripheral myeloid cells, albeit at lower numbers. Quite importantly, even DAM population may include heterogeneous subpopulations with distinct features (Barclay et al., 2024; Deczkowska, 2024).

Mutations in the human *GRN* (granulin) gene are a major cause of frontotemporal lobar degeneration (FTLD). To investigate mechanisms underlying FTLD-GRN, single-cell transcriptomics in the thalamus and frontal cortex of *Grn*^−/−^ mice and FTLD-GRN patients identified a conserved astrocytic pathology marked by upregulation of *GJA1, AQP4*, and *Apoe*, and downregulation of the glutamate transporter *SLC1A2*. These changes were associated with widespread synaptic degeneration in both species (Marsan et al., 2023).

Gazestani et al. (2023) generated a single-nucleus atlas from a rare cohort of cortical biopsies from living individuals with varying levels of AD pathology. Through integrative cross-disease and cross-species analysis, they identified a conserved set of cell states characteristic of early AD, termed the early cortical amyloid response. This included a transient hyperactive state in excitatory neurons that preceded their loss, validated by acute slice electrophysiology. Microglia exhibiting neuroinflammatory signatures expanded with disease progression (such as *GPNMB, THEMIS, PVALB*, and *WIF1*). Notably, both oligodendrocytes and pyramidal neurons upregulated genes involved in Aβ production and processing during this early phase (Gazestani et al., 2023).

In ALS, spatial and single-cell/single-nucleus transcriptomic approach was adopted to the tissues from ALS patients; and microglial involvement and transition toward disease-associated cell states were suggested (Maniatis et al., 2019; Masrori et al., 2025). A diminished response of astrocytes with *C9orf72* hexanucleotide repeat expansion provided a link of dysregulated ligand–receptor pairs in microglia and astrocytes (Masrori et al., 2025). Exome sequencing of ALS and rare-variant analyses identified loss-of-function mutations in *TBK1* (encoding TANK-binding kinase 1) in 13 of 252 familial ALS pedigrees, but not in sporadic ALS (Freischmidt et al., 2015). Recent finding in *TBK1* deficiency mice suggested that loss of *TBK1* in microglia causes an aging-like inflammatory state in these cells. Without any memory loss and learning deficits, microglial *TBK1* deficiency leads to social recognition impairments in mice at 4 months of age, resembling FTD symptoms. Unavoidably, microglial TBK1-KO leads to focal microglial activation and both CD8^+^ and CD8^−^ T cells infiltration in the basal ganglia (Lenoel et al., 2025).

In human ALS motor cortex, microglia show increased expression of Iba1 and CD68 (i.e., macrophages and monocytes marker), with CD68 levels strongly correlating with TDP-43 pathology (Swanson et al., 2023). Two distinct microglial subpopulations, characterized by high L-ferritin expression, were enriched in ALS motor cortex. Similar microglial changes were seen in the ALS mouse model, with CD68 increasing first, followed by L-ferritin, after TDP-43 inclusions appeared (Swanson et al., 2023). Therefore, the findings are not completely consistent across species, likely because humans are long-lived while mice have much shorter lifespans, resulting in different disease time courses and inflammatory milieu.

### DAA and reactive astrocytes in metabolism alteration

Finally, in this section we will discuss alterations in glycolytic metabolism and mitochondrial dysfunction in astrocytes. Elegant review studies are already published elsewhere (Jiang and Cadenas, 2014; Morita et al., 2019; Rahman and Suk, 2020; Bonvento and Bolaños, 2021), and we will try to summarize the conceptual advance concisely.

**Figure 4:**
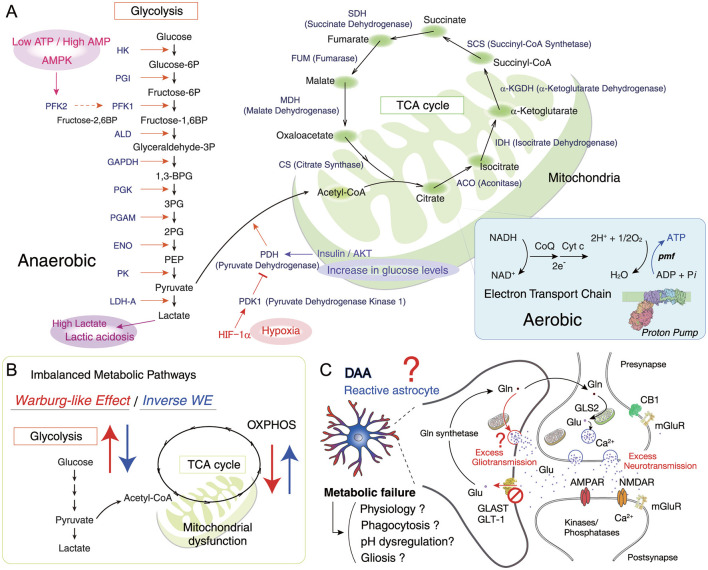
Metabolic dysfunctions of disease-associated and reactive astrocytes in stress insults. **(A)** Anaerobic glycolysis and mitochondrial oxidative phosphorylation. In-taken glucose is decomposed to pyruvate by different enzymes. Pyruvate is processed by pyruvate dehydrogenase (PDH) to acetyl-CoA, which enters the TCA cycle (i.e., citric acid cycle, Krebs cycle). Generated NADH releases hydrogen ions and transfers electrons to subsequent enzymes in the electron transport chain, CoQ and Cyt c. Electrons are used to generate water from protons, which generate an electrochemical gradient in the mitochondrial membrane. Using the proton-motive force (*pmf* ), ATP is generated. This process in the electron transport chain is aerobic. Low oxygen and ATP condition, excess lactate production, hypoxia, and glucose intake cause metabolic shift via different mechanisms. **(B)** Imbalanced metabolic pathways. In cancer, cells preferentially use “aerobic glycolysis.” Cancer cells convert glucose to lactate even in the presence of oxygen, which is called the Warburg effect, in general (Seyfried et al., 2025). In contrast, in some neurodegenerative conditions, astrocytes shift to glycolysis, producing lactate; neurons, however, show impaired OXPHOS (the Inverse Warburg effect; Demetrius and Simon, 2012; Demetrius et al., 2015). Note that a neuron model in neurodegeneration is shown, but not any astrocytes and lactate shuttle. **(C)** Upon metabolic dysfunctions of DAA and reactive astrocytes, physiological mechanisms such as excess glio-transmission, cellular phagocytotic promotion, and gliosis (fibrilization) are not yet elucidated. AMPK, AMP-activated protein kinase; HK, hexokinase; PGI, phosphoglucose isomerase; PFK1/2, phosphofructokinase-1/2; ALD, aldolase; GAPDH, glyceraldehyde-3-phosphate dehydrogenase; PGK, phosphoglycerate kinase; PGAM, phosphoglycerate mutase; ENO, enolase; PK, pyruvate kinase; LDH-A, lactate dehydrogenase A; 1,3-BPG, 1,3-bisphosphoglycerate; 2PG, 2-phosphoglyceric acid; 3PG, 3-phosphoglyceric acid; PEP, phosphoenolpyruvate; ADP, adenosine diphosphate; ATP, adenosine triphosphate; CoQ, Coenzyme Q; Cyt c, Cytochrome c; NADH, Nicotinamide adenine dinucleotide (*reduced form*); NAD^+^, nicotinamide adenine dinucleotide (*oxidized form*); HIF, hypoxia inducible factor; *pmf* , proton-motive force; TCA cycle, tricarboxylic acid cycle; OXPHOS, oxidative phosphorylation; Gln, glutamine; Glu, glutamate; GLS2, glutaminase 2; GLAST, glutamate aspartate transporter (also EAAT1); GLT-1, glutamate transporter 1 (also EAAT2); AMPAR, α-amino-3-hydroxy-5-methyl-4-isoxazolepropionic acid receptor; NMDAR, N-methyl-D-aspartate receptor; CB1, cannabinoid receptor 1; mGluR, metabotropic glutamate receptor.

Studies have shown that in various neurodegenerative conditions, DAAs or reactive astrocytes exhibit increased glycolytic activity alongside impaired mitochondrial oxidative phosphorylation (Jiang and Cadenas, 2014; Horvat et al., 2021; Pamies et al., 2021; Traxler et al., 2022; Acevedo et al., 2023; Theparambil et al., 2024). These metabolic switches should influence astrocyte functions such as energy supply, redox balance, and inflammatory responses, thereby contributing to disease progression (Figure 4). Patients with ALS often show impaired glucose tolerance (Harno et al., 1984) and chronically poor nutritional status (Kasarskis et al., 1996). The astrocytic glutamate transporter GLT-1/EAAT2 expression is markedly reduced (Rothstein et al., 1995). In AD, glucose transporter GLUT1 and GLUT3 expression is decreased in the brain (Simpson et al., 1994). Together, these clinical findings suggest that glial metabolic dysfunction may contribute to age-related neurodegenerative disorders.

In Figure 4A, we display anaerobic glycolysis and mitochondrial oxidative phosphorylation with the electron transport chain as the aerobic process. Metabolic shifts are known via different mechanisms. Under low oxygen and ATP condition, AMPK is activated and thereby stimulates glycolysis (Marsin et al., 2000). Excess lactate produced by enhanced glycolysis can lead to lactic acidosis in the brain (Magistretti and Allaman, 2018). HIF-1 promotes a metabolic shift in hypoxia by inducing PDK1, which inhibits pyruvate entry into the TCA cycle and redirects glucose metabolism to glycolysis to sustain ATP and reduce ROS (Kim et al., 2006; Semenza, 2010). High glucose intake can activate PDH indirectly through insulin (Jarett and Seals, 1979; Karwi et al., 2020; Figure 4A).

In cancer cells, “aerobic glycolysis” is preferentially used. Cancer cells convert glucose to lactate even in the presence of oxygen, which is called Warburg effect (Seyfried et al., 2025). In contrast, in some neurodegenerative conditions, astrocytes shift to glycolysis, producing lactate that neurons use for energy via lactate shuttle. Neurons, however, show impaired oxidative phosphorylation (OXPHOS). This metabolic coupling is called “Inverse Warburg effect” because, unlike cancer cells, neurons are energy-deficient despite oxygen, contributing to neurodegeneration (Demetrius and Simon, 2012; Demetrius et al., 2015). In neurodegeneration, the Warburg-like effect is a shift to inefficient aerobic glycolysis due to mitochondrial dysfunction, leading to energy failure (Figure 4B). These hypotheses are important for understanding metabolic and physiological consequences, and various transcriptomic and proteomic changes associated with psychiatric disorders, neurodegenerative diseases, and aging will help elucidate the precise mechanisms of biochemical alterations within and out of the organelle.

At present, glycolytic metabolism alterations and mitochondrial dysfunction of DAA or reactive astrocytes were reported in the contexts of (1) glycolytic metabolism alteration; and (2) mitochondrial dysfunction, leading to neuroinflammation, impaired neuroprotection, and age-related disease progression (Jiang and Cadenas, 2014; Horvat et al., 2021; Pamies et al., 2021; Traxler et al., 2022; Acevedo et al., 2023; Theparambil et al., 2024).

#### (1) Glycolytic metabolism alterations

##### Enhanced glycolysis

In disease states, astrocytes often shift their metabolism toward aerobic glycolysis (Warburg-like effect), increasing glucose uptake and lactate production even in the presence of oxygen. This metabolic reprogramming supports rapid ATP generation to meet increased energy demands during stress or inflammation (Fernández-Moncada et al., 2024; Theparambil et al., 2024), related to depression-like behavior and cognitive impairments in mice (Yao et al., 2023; Fernández-Moncada et al., 2024).

##### Lactate shuttle

The lactate produced by astrocytes can be exported and used as an alternative energy substrate by neurons (Mächler et al., 2016), supporting their survival under pathological conditions (Inverse Warburg effect). This astrocyte-neuron lactate shuttle is crucial for maintaining neuronal energy homeostasis when mitochondrial function is impaired (Suzuki et al., 2011; Chen et al., 2023).

##### Pentose phosphate pathway (PPP) activation

Increased glycolytic flux diverts glucose-6-phosphate into the PPP, which generates NADPH. NADPH is essential for maintaining cellular redox balance by regenerating reduced glutathione (GSH), a major antioxidant (Nóbrega-Pereira et al., 2016; Del Prado et al., 2024; Bar et al., 2025). This protects both astrocytes and neighboring neurons from oxidative damage.

#### (2) Mitochondrial dysfunction in DAAs and reactive astrocytes

##### Impaired oxidative phosphorylation (OXPHOS)

DAAs exhibit reduced mitochondrial respiratory chain activity, leading to decreased ATP production via OXPHOS. This forces astrocytes to rely more on glycolysis for energy, contributing to metabolic reprogramming (Liddelow et al., 2017; Habib et al., 2020; Polyzos et al., 2019; Guttenplan et al., 2020a).

##### Increased reactive oxygen species (ROS)

Dysfunctional mitochondria produce excessive ROS, which can damage mitochondrial DNA, proteins, and lipids, exacerbating mitochondrial impairment and triggering pro-inflammatory signaling (Vicente-Gutierrez et al., 2019; Mi et al., 2023; Tomasello et al., 2024). Astrocytes can release functional mitochondrial particles via a calcium- and CD38-dependent manner, which are then taken up by adjacent neurons after stroke, promoting neuronal survival in mice. Inhibition of this transfer worsens outcomes, suggesting a glia-to-neuron mitochondrial rescue pathway (Hayakawa et al., 2016).

##### Lipid metabolism and lipid droplets

Mitochondrial dysfunction impairs β-oxidation of fatty acids, leading to lipid droplet accumulation within astrocytes. This is associated with an inflammatory phenotype and can promote neurodegeneration. We will discuss this topic in the later section.

##### Calcium dysregulation

Mitochondrial defects disrupt calcium buffering in astrocytes, altering intracellular calcium signaling that affects neurotransmitter release, gliotransmission, and inflammatory responses (Motori et al., 2013; Habbas et al., 2015; Shah et al., 2022; Popov et al., 2023).

Upon metabolic dysfunctions of DAA and reactive astrocytes, it is elusive how physiological consequences are related in such as excess gliotransmission, cellular phagocytotic promotion, and gliosis (fibrilization). While basic research focuses on animal models, there are serious claims that those studies hardly reflect human diseases from pharmacological and human applications (Van Norman, 2019). Metabolic alterations in DAAs and reactive astrocytes promote the release of pro-inflammatory cytokines and chemokines, amplifying neuroinflammation. At a cellular mechanism, glutamate metabolism may be altered (Figure 4C), leading to excess gliotransmission. Such finding is observed in stress model (Habbas et al., 2015). Phagocytotic effect is assumed by complement pathways (Liddelow et al., 2017). In human neurological samples, α-ketoglutarate dehydrogenase activity was studied (Mastrogiacomo and Kish, 1994; Mastrogiacomo et al., 1993, 1996). Accordingly, gliosis may be via pathways related to α-ketoglutarate. Reduced mitochondrial ATP production and altered redox balance limit astrocytes' ability to support neuronal health and detoxify harmful substances. Therefore, the combined metabolic and functional disruptions in DAAs and reactive astrocytes exacerbate neuronal dysfunction, cellular pathophysiology, and cell death, contributing to progression in CNS pathology. Further studies are required.

Until here, we described transcriptomic, metabolic, and proteomic heterogeneity in reactive glial cells and their physiological relevance. As discussed, microglial research has moved past old two-part labels like “resting vs. activated” and “M1 vs. M2.” Transcriptomic and proteomic analyses unveiled diverse and complex microglial profiles; however, there is a risk of oversimplifying their roles by linking states too rigidly to functions. To better capture microglial diversity, we are required to clarify states, identity, and nomenclatures, depending on the context of development, sex, species, disease, and aging (Paolicelli et al., 2022). Similarly, astrocytes are recognized to change their shape, behavior, and gene activity in response to injury, disease, or infection in CNS. Although such response of reactive astrocytes was first described over 100 years ago, there are still many questions and debates remain in the contexts of diseases, recovery, and aging. The problems with labeling reactive astrocytes in simple categories, like “good vs. bad,” “neurotoxic vs. neuroprotective,” or “A1 vs. A2” should be redefined by measuring many molecular and functional features—ideally in living systems (Escartin et al., 2021). Based on the recent findings from comprehensive and multiscale studies, understanding of the glial heterogeneity is widely accepted and shared not only in microglia and astrocytes but also oligodendrocytes and even neurons (Kenigsbuch et al., 2022; Park et al., 2023). Therefore, transcriptomic heterogeneity has reshaped our understanding of cellular identity, and such plasticity appears to be a ubiquitous feature across diverse biological contexts. Organelles, metabolism, sex differences, and the epigenome are critical gateways to future discoveries in neuroscience, while clinical investigations require a deep understanding of the biology underlying spatial pathology.

## Cellular senescence

“*Evolutionary considerations suggest aging is caused not by active gene programming but by evolved limitations in somatic maintenance, resulting in a build-up of damage.”* (Kirkwood, 2005)

In this section, we will discuss important notions in cellular senescence and highlight selected recent findings, related to glial heterogeneity and disease association. While aging causes a gradual decline of physiological functions, cellular senescence is defined as a state of permanent “cell cycle arrest,” accompanied by distinct metabolic activity (Kuilman et al., 2010; Gorgoulis et al., 2019). These processes are closely linked, as aging leads to an accumulation of cellular senescence. This review focuses specifically on the latter.

Several distinct populations of microglia and macrophages have been described in the aging brain, including dark microglia (Bisht et al., 2016), IFN-responsive microglia (Kaya et al., 2022), and senescent-like microglia (Gross et al., 2025), underlying the heterogeneity of immune cell responses to aging-related stress. Dark microglia are characterized by an electron-dense cytoplasm under electron microscopy. Although rare under physiological conditions, their numbers increase with age and are remarkably elevated in neurodegenerative disorders (Bisht et al., 2016). IFN-responsive microglia have been reported to localize near CD8^+^ T cells in the aging white matter (Kaya et al., 2022). Senescent-like microglia, in contrast, limit remyelination through SASP that involves the release of multiple inflammatory mediators, including CCL11, CXCL9, and CCL5, and increased expression of IL-2, leading to impaired remyelination in mouse models (Gross et al., 2025). In a related context, lipid-associated macrophages have been shown to undergo an inflammatory transition during human atherosclerosis, potentially contributing to ischemic heart disease, stroke, and peripheral arterial disease (Dib et al., 2023).

Here, we review the heterogeneity of glial cells and glioinflammatory responses in different contexts linked to cellular senescence.

### Genomic instability

Before discussing cellular mechanism, we briefly introduce an important notion of genomic alteration in senescence. López-Otín et al. (2023) proposed twelve hallmarks of aging: genomic instability, telomere shortening, epigenetic alterations, impaired protein homeostasis, defective autophagy, deregulated nutrient-sensing, mitochondrial dysfunction, cellular senescence, stem cell exhaustion, altered intercellular communication, chronic inflammation, and microbial imbalance. These hallmarks are interconnected and tightly related to spatial compartmentalization, maintenance of homeostasis, and adequate responses to stress. Genome integrity and stability are continuously challenged by endogenous and environmental stress, causing diverse DNA lesions and genetic variation within tissues implicated in aging. Such genomic instability is accelerated and induces progressive decline with age, although organisms deploy nuclear and mitochondrial DNA repair systems (Kuilman et al., 2010; López-Otín et al., 2023).

In fact, genomic instability and inflammation are distinct hallmarks of aging, but the connection between them has not been completely understood. Recently, Miller et al. (2025) investigated a mechanism linking genomic instability and inflammation in senescent cells. They reported that p53 suppresses accumulation of cytoplasmic chromatin fragments (CCF), including DNA damage signaling marker γH2A.X, and its downstream inflammatory phenotype (e.g., SASP) of monocytes and macrophages in liver. p53 activation enhanced DNA repair, genome integrity, and suppressed CCF formation. Pharmacological inhibition of MDM2, leading to p53 activation in aged mice, reversed transcriptomic signatures of aging and age-associated accumulation of monocytes and macrophages. Mitochondrial ablation in senescent cells suppressed CCF formation and activates p53, suggesting that mitochondria-dependent formation of γH2A.X and CCF dampens nuclear DNA damage signaling and p53 activity in the tissue (Miller et al., 2025).

### Cell cycle arrest and chronic inflammation

Senescent cells are characterized by irreversible “cell cycle arrest,” triggered by senescence-associated stressors, including DNA damage responses (DDRs) induced by oxidative stress, ROS, smoking, poor nutrition, oncogenes, gut microbiota, and mitochondrial dysfunction. Chronic inflammation driven by infection and necrotic cellular debris exposure also contribute to senescence (Gorgoulis et al., 2019; Campisi, 2005; Kirkwood, 2005; Kuilman et al., 2010). Telomeres, which cap the ends of chromosomes, progressively shorten upon each round of cell division. The telomere shortening limits proliferative capability and drives senescence.

Campisi (2005) proposed that specific gene upregulated during senescence and proteins translated from these genes may alter cellular functions and tissue structures. More recently, research found that senescent cells secrete variety of bioactive molecules, including inflammatory cytokines, chemokines, growth factors, matrix metalloproteinases (MMPs), and so on. Those factors are collectively termed the senescence-associated secretory phenotype, SASP (Coppé et al., 2008; Gorgoulis et al., 2019). According to Ma and Farny (2023), senescence phenotypes in post-mitotic neurons cellular senescence are pronounced in enhanced activity of senescence-associated beta-galactosidase (SA-β-gal), cell cycle arrest [P53-P21 pathway and P16 pathway integrate to RB (retinoblastoma) hypophosphorylation], DNA damage, elevated RB hypophosphorylation, SASPs, and also senescence-associated heterochromatic foci (SAHFs), promoting cell cycle arrest (Ma and Farny, 2023; Saez-Atienzar and Masliah, 2020). As will be discussed later, SAHF play key roles in maintaining the senescent state, suppressing tumorigenesis, and influencing cell fate. However, their presence and formation patterns vary depending on the cell type and the specific senescence-inducing stimuli. p16^INK4a^ is a cell cycle inhibitor that blocks CDK4/6 activity, preventing cells from progressing from the G1 phase to the S phase of the cell cycle. This halts cell division and promotes cellular arrest. p16^INK4a^ is widely recognized as a biomarker of cellular senescence (Omori et al., 2020).

Therefore, the p53-p21 and p16-pRB tumor suppressor pathways are canonical to cellular senescence. While they prevent uncontrolled cell proliferation and cancer in youth, they contribute to tissue dysfunction and chronic inflammation with age. This dual role, known as antagonistic pleiotropy, beneficially suppresses tumor initiation early in life but detrimentally increases the risk of age-related pathologies, later.

## SASP in disease-associated glial and immune cells

Although senescent cells have ceased dividing, they are far from being “completely silent.” On the contrary, they remain metabolically and transcriptionally active, actively secreting proinflammatory molecules. SASP factors are secreted through both paracrine and autocrine, inducing the NF-κB pathway, inducing senescence in neighboring cells and promoting inflammation. As a result, SASP can propagate widely and contribute to tissue dysfunction, including chronic inflammation, fibrosis, and the progression of neurodegenerative diseases (Acosta et al., 2013; Nelson et al., 2012).

Recent studies suggest that senescent glial cells also produce SASP-like molecules. For example, senescent astrocytes show upregulated expression of IL-6, IL-8, IL-1β, MMP3, MMP10, and TIMP2, while neuroprotective factors such as IL-10, NGF (nerve growth factor), and BDNF (brain-derived neurotrophic factor) are downregulated (Lye et al., 2019). These changes shift astrocyte function toward a pro-inflammatory phenotype to downregulation of neuroprotective properties. Some studies also report increased p53 activation and decreased expression of Δ133p53α, a regulatory isoform of p53 (Turnquist et al., 2016). In AD, tau pathology correlates with oxidative stress exposure, leading to DNA damage, activation of p53, and the inflammasome. Notably, upregulation of serpinA3N was found in AD model mice (Han et al., 2024). These findings suggest that restoring Δ133p53α expression or inhibiting serpinA3N may serve as potential therapeutic targets of senescence astrocytes in neurodegeneration. Another study in ALS also indicates an important role in neurodegeneration (Maor-Nof et al., 2021).

Microglial senescence is predominantly detected in DAM, which appear in aging and neurodegenerative diseases. In senescence microglia, lactate concentration is elevated, and IκBα, a negative regulator of NF-κB, is phosphorylated and subsequently degraded, leading to activation of the NF-κB pathway (Wei L. et al., 2023; Li et al., 2025). As a result, senescent microglia secrete a range of SASP components, IL-6, IL-8, MMP3, MMP12, CXCL1, CXCL2, and CXCL10 (Cook et al., 2022). Transglutaminase 2 (Tgm2) covalently cross-links IκBα, further promoting NF-κB nuclear translocation and enhancing the expression of phosphorylated p53 and p21. BAY 11-7082, an NF-κB inhibitor, reduced IL-6 expression, while Cys-D, a Tgm2 inhibitor, suppresses NF-κB nuclear transport, both emerging as potential therapeutic candidates for senescent microglia (Cook et al., 2022).

Senescent microglia are considered a component of immunosenescence. We also briefly address senescence in other immune cell types. Senescent T cells exhibited lack in production of IL-2, and IL-4, while secreting high levels of IL-6, IL-8, osteopontin (OPN), IFN-γ, CCL3, and CCL4 upon T cell receptor (TCR) stimulation (Shimatani et al., 2009; Minato et al., 2020; Angelova and Brown, 2018). These cells, termed senescence-associated T (SA-T) cells, have been proposed to express SPP1, the gene encoding OPN, as a potential marker of age-related T cell senescence (Minato et al., 2020). Senescent macrophages, including bone marrow-derived macrophages exposed to high-dose radiation, also secrete IL-1α, IL-6, TNF-α, MCP-1, CCL2, CXCL10, CCL17, MMP2, MMP9, and MMP12 (Su L. et al., 2021; Smith et al., 2024). Aging impaired phagocytosis in tissue-resident peritoneal macrophages but not in bone marrow-derived macrophages or monocytes (Linehan et al., 2014). The decline of macrophage phagocytosis was linked to changes in the aged tissue microenvironment, including increased immune cells and IL-10 from B cells. In short, senescent glial and immune cells produce distinct SASP profiles, which may serve as both mechanistic markers and therapeutic targets in aging and neuroinflammatory disorders.

## Relationship between brain lipid metabolism and senescence

### The role of lipids in the brain

Lipids constitute approximately 60% of the brain's dry mass, with a marked concentration within neuronal membranes and the myelin sheath, which functions as a critical insulator for axonal conduction. Beyond serving as structural components through the lipid bilayer, lipids participate in diverse cellular processes including the modulation of intracellular and intercellular signaling pathways, energy storage, and the synthesis and maintenance of myelin.

The smooth endoplasmic reticulum (SER) is the organelle that produces lipid in cells. Lipid biosynthesis is an energy-intensive metabolic process; therefore, when ATP production is impaired due to mitochondrial dysfunction (Figure 4), lipid synthesis is consequently prevented. Mitochondrial impairment profoundly affects lipid production and maintenance by disrupting energy supply and altering lipid metabolism. Aging engenders substantial remodeling of cerebral lipid profiles. A notable decline in polyunsaturated fatty acids (PUFAs) occurs alongside an accumulation of lipids susceptible to oxidative damage. These compositional shifts undermine membrane fluidity and integrity, thereby impairing neuronal function. Concurrently, increased production of ROS exacerbates lipid peroxidation, compromising membrane architecture and promoting myelin degradation. Age-related attenuation of enzymes governing lipid metabolism disrupts the equilibrium between lipid synthesis and catabolism. Given the lipid-rich composition of myelin, such metabolic perturbations precipitate myelin deterioration and demyelination, leading to a decrease in nerve conduction velocity (Johnson and Stolzing, 2019; Chung, 2021).

Pathological lipid metabolism and increased oxidative stress are implicated in the etiology of neurodegenerative diseases, including AD and Parkinson's disease (PD). APOE, primarily synthesized by astrocytes and secreted as HDL-like particles, facilitates lipid trafficking via low-density lipoprotein receptor (LDLR)-mediated uptake. The APOE4 isoform is a major genetic risk factor for late-onset AD, whereas APOE2 exerts a protective effect as explained earlier. Dysregulated lipid metabolism also promotes aberrant synthesis of pro-inflammatory lipid mediators such as prostaglandins and leukotrienes, thereby sustaining chronic neuroinflammatory states (Johnson and Stolzing, 2019; Chung, 2021). In AD, to investigate APOE4—the strongest genetic risk factor for AD, Tcw et al. (2022) examined the effects of human-specific, APOE4-driven lipid metabolic regulation. Global transcriptomic analyses revealed APOE4-specific dysregulation of lipid metabolism in astrocytes and microglia, with human-specific features. In astrocytes, APOE4 increased *de novo* cholesterol synthesis despite intracellular cholesterol accumulation caused by lysosomal sequestration. Matrisome dysregulation in astrocytes co-cultured with neurons was linked to elevated chemotaxis, glial activation, and lipid biosynthesis, mirroring altered matrisome signaling observed in human brain tissue (Tcw et al., 2022).

## Liquid–liquid phase separation in the brain glia

Liquid–liquid phase separation (LLPS) in synaptic function, neurodegenerative diseases, and aging has been extensively reviewed elsewhere (Hyman et al., 2014; Su Q. et al., 2021; Wang et al., 2021; Milicevic et al., 2022). Briefly, LLPS is a biophysical process whereby biomolecules such as proteins and RNA spontaneously demix to form dynamic, liquid-like condensates within the cellular milieu, establishing functional membrane-less compartments (Li et al., 2018; Garcia-Pardo and Ventura, 2024). LLPS underpins critical neuronal processes including local translation, RNA metabolism, and stress response. LLPS occurs in glial cell types, including astrocytes, microglia, and oligodendrocytes, where its dysregulation is implicated in neuroinflammation, demyelination, and the pathogenesis of neurodegenerative diseases.

The disruption of LLPS in the brain primarily is considered to stem from several factors: (1) mutations in proteins or expansions of low-complexity domains that favor pathological aggregation; (2) age-related decline and chronic stress-mediated impairment of protein quality control pathways; (3) aberrant interactions with RNA species that alter condensate biophysical properties; (4) dysregulation of post-translational modifications governing protein phase behavior; and (5) localized protein overaccumulation resulting in uncontrolled LLPS.

Aging is accompanied by deteriorating proteostasis and altered modification landscapes, which compromise the reversible and dynamic property of LLPS, triggering the formation of aberrant, solid-like aggregates that exacerbate neuronal dysfunction and neurodegeneration. While evidence directly linking LLPS dysfunction to psychiatric disorders remains nascent, abnormalities in RNA metabolism and dysregulated local translation linked to LLPS mechanisms have been suggested. Dysfunctional LLPS-associated proteins (e.g., FMRP, TIA1, G3BP1) and aberrant stress granules have been implicated in psychiatric disorders such as autism spectrum disorder, schizophrenia, and depression (Darnell et al., 2011; Clifton et al., 2021; Jia et al., 2022; Mackenzie et al., 2017), though complete causal relationships await further elucidation. Given that glial and neuronal cells undergo aging-related changes, including SASP, alterations in extracellular matrix composition, mitochondrial DNA release, and activation of inflammatory signaling pathways, shifts in membrane or lipid environments may influence these processes by modulating LLPS and domain dynamics.

In neurodegenerative diseases, aberrant LLPS is observed, and normal LLPS regulation is disrupted. In this context, droplets that are normally reversible may transition into irreversible aggregates or have fibrogenic properties, leading to reactive glia. These droplets also harden and lose their dynamic properties. Eventually, functional droplets transform into abnormal structures with toxic properties, especially in neurodegeneration. For instance, FUS, TDP-43, and TIA1 form aggregates and aberrant stress granules in ALS; TDP-43 and hnRNPA1 promote the formation of solid or gel-like structures, leading to cytotoxicity in FTD; Tau, α-synuclein, and FMRP undergo fibrillization via LLPS in AD (Pakravan et al., 2021; Naskar et al., 2023).

Importantly, Gasset-Rosa et al. (2019) reveals that TDP-43 phase separation is not just a biophysical occurrence, but a central pathological mechanism in ALS and FTD, addressing the view of aggregation as a passive consequence to a dynamic and stress-induced process with functional consequences on nuclear transport and cellular survival. Transient stress or increased cytoplasmic TDP-43 triggers phase separation into toxic cytoplasmic droplets that block nuclear import, deplete nuclear TDP-43, and cause cell death, offering a present mechanistic model for ALS/FTD pathology (Gasset-Rosa et al., 2019). Meanwhile, stress granules are small cytoplasmic assemblies, formed in response to cellular stress, enabling essential transcription. Phosphorylated TDP-43 immunoreactivity correlates with stress granule assembly in neurons and glia, caspase-3 activation, and neurodegeneration (Yan et al., 2025). Through LLPS, stress granule aggregation interrupts translation and sequesters untranslated mRNAs, as demonstrated by super-resolution microscopy, thereby prioritizing stress-responsive mRNA translation (Yan et al., 2025). In contrast, reducing lipid droplet accumulation in brain macrophages, especially microglia and border-associated macrophages (BAMs), restores their phagocytic function and limits Aβ pathology (Wu et al., 2025). Lipid droplets (LDs) are energy-storing organelles found in all cells. In AD, CD11c^+^ microglia and CD206^+^ BAMs accumulate LDs, particularly near Aβ plaques. And these cells express lipid-associated genes (e.g., *Trem2, Lpl, Apoe*). Lipid droplets-loaded microglia, termed LDAM, are dysfunctional. LDAM reduced phagocytosis of Aβ and apoptotic neurons. Production of pro-inflammatory cytokines and ROS were increased. Targeting FIT2, a protein essential for LD formation, the authors demonstrated reducing LD accumulation in brain macrophages could restore their pathological phenotypes in an AD mouse model (Wu et al., 2025).

## Heterogeneity upon the origin and glial senescence

### Yolk-sac-derived and bone marrow-derived

Microglia are essential for immune defense and brain homeostasis. Their roles extend beyond neuronal development and synaptic formation to include contributions to disease progression and aging. Studies have shown that microglia originate from embryonic yolk sac progenitors during early development, are of primitive hematopoietic origin, and persist into adulthood, maintaining independence from bone marrow-derived myeloid cells throughout life, shaping their distinct functions across maturation and aging (Ginhoux et al., 2013; Stremmel et al., 2018; Carrier et al., 2024; Weinhaus et al., 2025). Recently, Weinhaus et al. (2025) investigated differential regulation of fetal bone marrow and liver hematopoiesis by yolk-sac-derived myeloid cells and identified a VCAM1^+^ sinusoidal colonization niche in the diaphysis that regulates neutrophil and hematopoietic stem cell colonization of the bone marrow (Weinhaus et al., 2025). However, as we discussed DAM and DAA, it remains largely unclear how reactive astrocytes, which originate from the neuroectoderm, acquire functional similarities, such as inflammatory responses and phagocytic activity, to immune cells like microglia and macrophages that arise from primitive hematopoietic progenitors and mesoderm-derived bone marrow hematopoietic cells. In the final section, we will discuss epigenomes causing glial heterogeneity and possible association with glial senescence.

### Epigenomes causing glial heterogeneity

**Figure 5:**
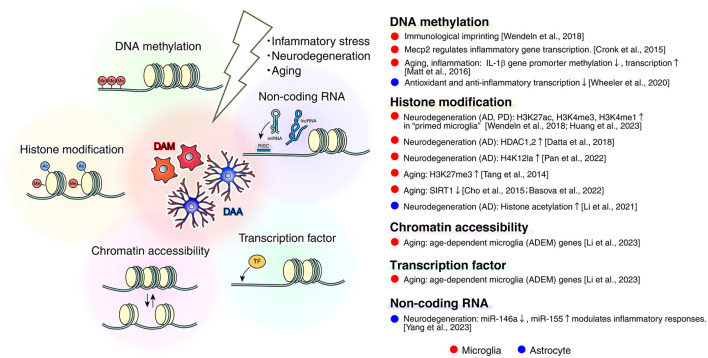
Epigenetic modifications of microglia and astrocytes in neurodegenerative diseases. *DNA methylation* is the addition of a methyl group to DNA cytosine bases, typically at CpG sites. This process can silence gene expression without altering the DNA sequence, in development, cellular differentiation, and disease regulation. *Histone modifications* involve chemical tags by acetylation, methylation, and phosphorylation on histone proteins around which DNA is wrapped. These modifications alter chromatin structure and influence gene expression without changing the DNA sequence. *Chromatin accessibility* refers to how open or closed the chromatin structure is, which influences whether transcription factors can bind to DNA and controls gene expression. This epigenetic mechanism is regulated by histone modifications and chromatin remodeling complexes. *Transcription factors* regulate gene expression not only directly but also by recruiting epigenetic modifiers such as histone acetyltransferases or chromatin remodeling complexes. Some transcription factors can access closed chromatin and initiate epigenetic changes that open chromatin and enable gene transcription. *Non-coding RNAs*, especially long non-coding RNAs (lncRNAs) and microRNAs (miRNAs), can recruit chromatin-modifying complexes to specific genomic regions or silence gene expression post-transcriptionally, thereby influencing chromatin structure and gene activity.

The term “epigenetics” originally described how interactions between the genome and the environment influence development and differentiation in complex organisms (Figure 5). Chromatin is a complex of DNA and nuclear proteins, primarily histones, organized into structural units called nucleosomes. Each nucleosome consists of 147 base pairs of DNA wrapped around an octamer of histone proteins, two H3-H4 dimers flanked by two H2A-H2B dimers, with histone tails extending outward into the nucleus. Histone H1 binds to linker DNA between nucleosomes, influencing chromatin compaction. The spacing of nucleosomes defines chromatin architecture, euchromatin or heterochromatin. Both DNA and histone tail modifications regulate gene accessibility and transcriptional activity. Chromatin structure and gene accessibility to transcriptional machinery are regulated by modifications to both DNA and histone tails (Handy et al., 2011; Houston et al., 2013; Li Y. E. et al., 2023).

Those epigenetic tags of DNA methylation and histone acetylation enable acquisition, maintenance, and inheritance of gene regulation. In the following paragraphs, we like to introduce recent epigenomic findings of microglia regarding to (a) DNA Methylation; (b) Histone Modifications; (c) Chromatin Accessibility; (d) Transcription Factors and Epigenomic Interactions; and (e) Impact of Environment and Disease. These mechanisms coordinate to regulate cell-type specific gene expression, contributing to heterogeneity within glial populations. Microglial heterogeneity is formed by development, environment, and brain region, as well as states of activation (homeostatic vs. reactive microglia; Figure 5).

### Microglia epigenomics and heterogeneity

#### (a) DNA methylation

DNA methylation, primarily occurring at CpG sites, typically acts as a repressive tag for gene expression. In microglia-specific genes, such as *PU.1, Cx3cr1, P2ry12*, and *Tmem119*, distinct DNA methylation patterns contribute to the maintenance of microglial identity (Yeh and Ikezu, 2019). DNA methylation potentially changes during development and in response to inflammation can shift microglia toward disease-associated phenotypes. Reactivated microglia showed enrichment for the thyroid hormone signaling pathway, including a putative enhancer for HIF-1α (Wendeln et al., 2018). Mutations in methyl-CpG binding protein 2 (MeCP2), an epigenetic regulator, are the main cause of Rett syndrome. In Mecp2-null mice, glucocorticoid- and hypoxia-induced transcripts expression was increased in microglia and peritoneal macrophages. Mecp2 regulate inflammatory gene transcription in response to TNF stimulation. Restoring Mecp2 in microglia postnatally extends lifespan (Cronk et al., 2015).

#### (b) Histone modifications

Histone modifications, including methylation, acetylation, phosphorylation, and ubiquitination, can activate or repress genes depending on the mark and context. In glioblastoma, microglia are chronically exposed to immunosuppressive signals that support tumor progression. This “transcriptional memory” is driven by epigenetic changes, including histone deacetylation (mediated by histone deacetylases, HDACs) and repressive histone methylation (H3K27 trimethylation) at inflammatory gene loci (Maleszewska et al., 2021). Increased epigenetic marks (H3K27ac, H3K4me3, H3K4me1) were associated with primed-microglia in Parkinson's disease (Huang et al., 2023) and AD (Wendeln et al., 2018), and glycolysis/H4K12la/PKM2 positive feedback loop exacerbated microglial dysfunction (Pan et al., 2022).

#### (c) Chromatin accessibility

Chromatin remodeling changes nucleosome positioning to modulates DNA accessibility; thereby, 3D chromatin architecture influences enhancer-promoter interactions. Assays such as ATAC-seq and ChIP-seq have identified distinct open chromatin landscapes near the *BIN1* gene locus in both mouse and human microglia, marked by PU.1, H3K27ac, and H3K4me2. Loci close to *SPI1* (encoding PU.1), *P2RY12*, and *SALL1* function as environment-dependent enhancers, varying across different brain regions or disease states. Microglial enhancer regions, marked by H3K27ac, show dynamic changes that correlate with microglial heterogeneity (Gosselin et al., 2017). In the homeostatic state, hypomethylated regions are concentrated near immune-related genes, and undergo extensive reorganization in response to stimuli.

#### (d) Transcription factors and epigenomic interactions

Key microglial transcription factors (e.g., PU.1, IRF8) recruit chromatin remodeling and histone modification to establish cell-specific epigenomic landscapes. PU.1, in cooperation with C/EBP, plays a critical role in opening chromatin at microglial enhancers (Yeh and Ikezu, 2019; Troutman et al., 2021). Collaborative transcription factor binding of lineage-determining transcription factors (LDTFs) and dozens of co-expressed collaborative transcription factors (CTFs), enabling primed enhancer and poised-repressed enhancer (Troutman et al., 2021).

#### (e) Impact of environmental stress and diseases

Aging, neurodegeneration (e.g., Alzheimer's disease), and inflammation induce epigenetic reprogramming in microglia, leading to subpopulations with pro-inflammatory or neuroprotective phenotypes (Gosselin et al., 2017; Wendeln et al., 2018; Hayes et al., 2022). Epigenetic plasticity may underlie their ability to adopt diverse functional states.

### Astrocyte epigenomics and heterogeneity

Astrocytes are also heterogenous by brain region and physiological or pathological conditions, showing transcriptional, morphological, and physiological heterogeneity, as already described. We will discuss their epigenetic alterations (Figure 5).

#### (a) DNA methylation

DNA methylation is developmentally regulated; immature vs. mature astrocytes have distinct methylomes (Takizawa et al., 2001; Wheeler et al., 2020; He et al., 2020; MacArthur et al., 2024; Kremer et al., 2024) with an indispensable role of STAT3 activation due to methylation at a CpG site in the GFAP promoter (Takizawa et al., 2001), and in TET1/2/3-mediated DNA demethylation for establishing neural stem cell identity and enabling glial (e.g., astrocyte and oligodendrocyte) differentiation (MacArthur et al., 2024). While Tet2 promotes astrocyte differentiation from neural stem cells by demethylating astroglial genes, like *Gfap*, the transcription factor Olig2 suppresses Tet2 expression, thereby indirectly inhibiting astrocyte formation (He et al., 2020). Ischemic injury causes striatal astrocytes to acquire stem cell-like properties through methylome reprogramming, dependent on *DNMT3A* (Kremer et al., 2024). Astrocyte-specific genes (e.g., *Gfap, Aqp4, Mat2a, Mafg*, and *DNMT3B*) have unique DNA methylation profiles that stabilize astrocyte identity upon the inflammation (Wheeler et al., 2020). Demethylation of key loci can activate reactive astrocyte programs.

#### (b) Histone modifications

Active markers like H3K27ac signify astrocyte enhancers controlling region-specific genes (Welle et al., 2021; Park et al., 2022). Many histone modifications were unveiled, and there is an excellent review study already (Park et al., 2022). Repressive histone marks suppress genes from other lineages (neuronal, oligodendrocyte), maintaining astrocyte specificity in the context of embryonic neurogenesis, adult neurogenesis, neurodegenerative (e.g., HDAC2, HDAC6, CBP/p300, MLL1/3/4, SETD1A/B, SIRT1 and others in AD; CBP, HDACs, SIRT1 and others in PD), and psychiatric disorders (e.g., HDAC1, MLL1, SETD1A/B, GLP, G9a and others in SCZ; Park et al., 2022). Reactive astrocytes in injury show epigenetic remodeling, including increased H3K27ac at inflammatory genes (Lee et al., 2024).

#### (c) Chromatin accessibility

Single-cell ATAC-seq reveals diverse open chromatin profiles across astrocyte subtypes (Li Y. E. et al., 2023). Chromatin loops bring enhancers into proximity with promoters to regulate astrocyte subtype-specific genes, such as *NFIA, SOX9, RORB, LHX2*, and *FEZF2*. Transcription factors (Rorb, Dbx2, Lhx2, and Fezf2) drive mature gene expression, and 3D culture with FGF2 enhances their expression and astrocyte maturation (Lattke et al., 2021). Astrocytes are central to the molecular changes in tauopathies, with disease-specific chromatin accessibility linked to genetic risk variants (Briel et al., 2022). Hypomethylation at the promoters of genes involved in neural development and maturation.

#### (d) Non-coding RNAs and epigenomic interactions

Astrocyte heterogeneity is also modulated by microRNAs and lncRNAs that influence epigenetic states and gene expression. For example, miR-146a down, miR-155 up modulates inflammatory responses in astrocytes epigenetically in neurodegenerative diseases (Yang et al., 2023).

#### (e) Epigenetic response to environmental stress

Astrocytes exhibit epigenomic plasticity in response to CNS injury, infection, inflammation, and neurodegeneration. Epigenetic regulators like HDACs modulate reactive astrocyte phenotypes. Brain region-specific epigenomic landscapes also contribute to functional differences (e.g., cortical vs. spinal cord astrocytes; Li Y. E. et al., 2023).

### Possible heterogeneity of glial senescence

As discussed, cellular senescence is a stable form of cell cycle arrest triggered by stress, aging, or oncogene activation. Senescent cells undergo profound epigenomic changes that distinguish them from both proliferating and quiescent cells (Alshaebi et al., 2025). In this section, we briefly continue the discussion on the changes essential to establishing and maintaining the senescent phenotype of glial cells. In general, the epigenetic landscape of microglial aging is expected to include altered gene expression on (1) Global chromatin changes (e.g., formation of SAHF and loss of Lamin B1); (2) Histone modifications (repressive and activating marks, as well as histone loss and replacement); (3) DNA methylation changes (global and focal hypermethylation, and epigenetic drift); (4) Chromatin accessibility and genome architecture; (5) Role of chromatin-modifying enzymes (histone methyltransferases/demethylases and histone acetyltransferases/deacetylases); (6) Non-coding RNAs, miRNA, and epigenetic regulation; (7) DNA damage and chromatin response; and (8) Epigenetic memory and reversibility.

In line with the mechanisms described above, recent studies on the epigenetic regulation of microglial senescence can be summarized as follows (Figure 5): global DNA hypomethylation (Cho et al., 2015; Matt et al., 2016); histone modifications (H3K27ac, H3K4me3, H3K27me3, and JMJD3; Datta et al., 2018; Pan et al., 2022; Tang et al., 2014); age-associated increases in chromatin accessibility Li X. et al., (2023); and reduced SIRT1 expression accompanied by miRNA alterations (Cho et al., 2015; Basova et al., 2022). Aged microglia displayed DNA hypomethylation and interleukin-1 beta (IL-1β) promoter activation in BV cells (Matt et al., 2016). SIRT1 deficiency in aging microglia contributes to aging- or tau-mediated memory deficits by upregulating IL-1β through promoter-specific hypomethylation (Cho et al., 2015). Li X. et al. (2023) identified age-dependent microglia (ADEM) genes, including 57 and 14 ADEM genes positively correlated with age (P-ADEM genes) in female and male mice, respectively. In general, the chromatin accessibility of P-ADEM genes increased when microglia became aged, whereas negatively correlated ADEM (N-ADEM) genes showed reduced accessibility Li X. et al., (2023). AD model mice suggested an increase in Hdac1/Hdac2, glycolysis/H4K12la/PKM2 signaling, and a decrease in Jmjd3, a histone H3K27me3 demethylase (Datta et al., 2018; Pan et al., 2022; Tang et al., 2014). An inverse correlation between miR-142 and SIRT1 expression was observed in the brains of long-lived aged rhesus macaques (Basova et al., 2022).

In astrocytes, epigenetic regulations in their senescence remains under investigation (Labarta-Bajo and Allen, 2025), while several studies have reported the histone acetylation and increased H3K27ac enrichment in AD astrocytes Li X. et al., (2021), H2AFJ increase in AD induced-neurons (iNs; Herdy et al., 2022), and N6-methyladenosine (m6A) methyltransferase METTL3 regulation of the NEAT1/miR-377-3p/Nampt in mouse astrocytes in cerebral ischemia (Hu et al., 2024). Further research in both areas is ongoing.

Currently, senolytics and their potential to enhance longevity and promote cellular rejuvenation remain a subject of active debate (Bussian et al., 2018; Johmura et al., 2021; Wang et al., 2022; Meguro et al., 2024; Magkouta et al., 2025). To advance this field, more refined and tissue-selective strategies are under investigation, including minimally invasive delivery systems and prodrugs engineered to become active exclusively within senescent microenvironments. It is particularly notable that clearance of senescent cells prevents tau-dependent pathology and cognitive decline in neurodegeneration. Bussian et al. (2018) reported that removal of these cells in INK-ATTAC transgenic mice prevents gliosis, reduced tau hyperphosphorylation in both soluble and insoluble forms, impeded neurofibrillary tangle formation, and prevented cortical and hippocampal neurodegeneration, thus preserving cognitive function. Pharmacological intervention with a first-generation senolytic navitoclax (ABT263), a Bcl-2 inhibitor, modulated tau aggregation (Bussian et al., 2018) but had bone marrow toxicity (thrombocytopenia, low blood platelet count).

## Concluding remarks and perspectives

In this 2024 Neurophysiology topic review, we strived to summarize recent advances in the field of glioinflammation. Glial cells, particularly microglia and astrocytes, play essential roles in maintaining brain homeostasis and responding to pathological insults. Originally arising from the innate immune system, these cells have diversified significantly across animal phyla, resulting in remarkable heterogeneity in both function and phenotype. Recent research has revealed that glial cells are not only passive responders but also active modulators of psychiatric and neurodegenerative disorders. Stress-induced glial alterations during development can predispose individuals to psychiatric illnesses, while disease-associated glial phenotypes, such as DAM and DAAs, contribute to the progression of conditions like AD and ALS. DAM and DAA would be important conceptual entities, and disease association can be extended to various cell types, including other immune cells, glia, and neurons. Distinct stressors induce stress-related modulations that represent a conventional yet conceptually novel form of plasticity, which may have been both inherited and acquired through evolution and selection. Furthermore, cellular senescence and the SASP exacerbate chronic inflammation and glial dysfunction, with sex differences and lipid metabolism playing modulating roles. Metabolic alteration and epigenetic regulation and developmental origin further drive glial heterogeneity and aging trajectories. Understanding the mechanisms behind glial diversity and dysfunction, especially in the context of innate immunity signaling (e.g., cGAS-STING) in mitochondria, phase separation in cellular lipid, and senolytics progress, may unlock novel therapeutic avenues. Future research must navigate the complexity of glial states to precisely target disease-relevant glial subtypes while preserving physiological functions.

In the end, we put our perspectives alongside the neuroimmunology and glioimmunology field for the therapeutic strategies, including AI prediction of druggable chemicals.

### AI-driven drug discovery and protein design

Although it is beyond the topic of glial heterogeneity, we lastly introduce recent advances in artificial intelligence (AI) and it potential to drug discovery to close the review. Deep generative learning prediction of druggable chemicals provides rapid predictions of receptor binding compounds and antibiotics from database (Zhavoronkov et al., 2019; Stokes et al., 2020).

In the 2024 Nobel Prizes, significant contributions rooted in AI were recognized in both Physics and Chemistry, despite there being no dedicated Nobel prize category for AI. John J. Hopfield from the United States and Geoffrey Hinton from Canada were awarded for their foundational discoveries and inventions that made machine learning through artificial neural networks possible, laying the theoretical groundwork for much of modern AI. Meanwhile, David Baker of the University of Washington, along with Demis Hassabis and John Jumper from DeepMind in the UK, received the Chemistry Prize for their groundbreaking work in computational protein design and structure prediction. Their efforts, particularly the development of AlphaFold, have had a transformative impact on the life sciences, enabling remarkable advances in understanding protein structures at scale (Hopfield and Hinton, 2024; Baek et al., 2021; Jumper et al., 2021). Now, Alexander Rives and colleagues challenge the simulation of molecular evolution in 500 million years using generative programming (Rives et al., 2021; Lin Z. et al., 2023; Hayes et al., 2025). Scaling language models to 15 billion parameters enables direct prediction of atomic-resolution protein structures from primary sequences, dramatically accelerating high-resolution structure determination (Lin Z. et al., 2023). Leveraging vast evolutionary data, ESM3 is an advanced multimodal model that synthesizes functional proteins by jointly considering their sequence, structure, and function, excelling at complex prompt interpretation and alignment refinement. Impressively, it generated a bright fluorescent protein, esmGFP, sharing just 58% sequence identity with known proteins, representing an evolutionary gap of approximately 500 million years (Hayes et al., 2025).

### Integrating AI and multi-omics for understanding of glial heterogeneity

Nowadays, AI-driven multi-omic data integration approaches are increasingly employed for early disease diagnosis, biomarker discovery, and the development of personalized medicine (Nam et al., 2024; Wu and Xie, 2025; Ren et al., 2025; Bhushan and Misra, 2025). However, beyond simply applying these technologies within a specific field, it is crucial to emphasize the underlying purposes and scientific rationale guiding their use. The quality and relevance of the training data from limited datasets are essential for the success of these AI models. Generative AI approaches, such as GANs and diffusion models, may help but sophisticated estimation and validation models are required. The human genome encodes approximately 20,000 protein-coding genes, forming the foundation of multi-omic investigations. Advances in spatial transcriptomics now allow for the *in situ* profiling of transcriptomes encompassing ~20,000 genes within tissue samples, using platforms such as those from Illumina, 10x Genomics, and NanoString. Moreover, recent developments in spatial proteomics enable high-dimensional mapping of protein expression across tissue sections. Notable technologies include Hyperion (Standard BioTools), CosMx and GeoMx (NanoString), CellSpace (Bruker), PhenoCycler^®^-Fusion (Akoya Biosciences), and Microscoop^®^ Mint (Syncell), among others. Comprehensive multi-phenotypic analyses must incorporate not only pathophysiological characterization but also accurate human diagnostic data. Integrating epigenomic studies further advances our understanding of gene regulatory mechanisms. In parallel, metabolomics and lipidomics are poised to contribute valuable insights into cellular and systemic biochemical states. Additionally, cellular morphology, particularly of abundant glial cells, has already been analyzed using machine learning algorithms and automated segmentation methods (Hikosaka et al., 2025; Marini et al., 2025), highlighting the increasing role of computational approaches in modern biomedical research and their availability for highly dimensional information.

Implementing the aforementioned approaches may remain a formidable task due to constraints in both time and computational capacity. In the CNS, it is well recognized that an immense diversity of neurons alongside glial cell populations, with both lineages exhibiting significant cellular heterogeneity (Siletti et al., 2023; McKeever et al., 2025). Researchers recognize that transcriptomic data do not always align with proteomic signatures. However, integrating or predicting these relationships using AI-based approaches may open new avenues for elucidating previously unknown physiological and phenotypic aspects. We refer to these multi-omics integrative approaches as “physiolomics” (e.g., patch-sequencing, spatial RNA-seq following *in vivo* imaging) or “phenotomics” (e.g., behavioral profiling, brain activity mapping, and clinical or pathological diagnoses). While this review has primarily focused on the molecular and functional diversity of glial cells, neurons themselves notably encompass a wide spectrum of distinct cell subtypes, each contributing to the complexity of brain physiology and disease. Despite those obstacles, it should be very beneficial to elucidate the molecular trajectories that drive glial heterogeneity toward pathological states and to identify strategies for reverting, maintaining homeostatic status, and rejuvenating. In summary, generative AI and advanced deep learning frameworks are being incorporated into various scientific fields including biomedicine, which are reshaping our interpretation of existing data and enabling more precise predictions from large-scale datasets by multi-omics platforms and spatially high-resolution, comprehensive imaging systems.
